# *Paracoccidioides* spp. ferrous and ferric iron assimilation pathways

**DOI:** 10.3389/fmicb.2015.00821

**Published:** 2015-08-12

**Authors:** Elisa Flávia L. C. Bailão, Patrícia de Sousa Lima, Mirelle G. Silva-Bailão, Alexandre M. Bailão, Gabriel da Rocha Fernandes, Daniel J. Kosman, Célia Maria de Almeida Soares

**Affiliations:** ^1^Laboratório de Biologia Molecular, Instituto de Ciências Biológicas, Universidade Federal de GoiásGoiânia, Brazil; ^2^Laboratório de Bioinformática, Universidade Católica de BrasíliaBrasília, Brazil; ^3^Department of Biochemistry, School of Medicine and Biomedical Sciences, State University of New York at BuffaloBuffalo, NY, USA

**Keywords:** iron reduction, iron uptake, ferric reductase, zinc-regulated transporter, multicopper oxidases, paracoccidioidomycosis

## Abstract

Iron is an essential micronutrient for almost all organisms, including fungi. Usually, fungi can uptake iron through receptor-mediated internalization of a siderophore or heme, and/or reductive iron assimilation (RIA). Traditionally, the RIA pathway consists of ferric reductases (Fres), ferroxidase (Fet3) and a high-affinity iron permease (Ftr1). *Paracoccidioides* spp. genomes do not present an Ftr1 homolog. However, this fungus expresses zinc regulated transporter homologs (Zrts), members of the ZIP family of membrane transporters that are able in some organisms to transport zinc and iron. A 2,3,5-triphenyltetrazolium chloride (TTC)-overlay assay indicates that both *Pb*01 and *Pb*18 express a ferric reductase activity; however, ^59^Fe uptake assays indicate that only in *Pb*18 is this activity coupled to a reductase-dependent iron uptake pathway. In addition, Zrts are up-regulated in iron deprivation, as indicated by RNAseq and qRT-PCR using *Pb*01 transcripts. RNAseq strategy also demonstrated that transcripts related to siderophore uptake and biosynthesis are up-regulated in iron-deprived condition. The data suggest that the fungus could use both a non-classical RIA, comprising ferric reductases and Fe/Zn permeases (Zrts), and siderophore uptake pathways under iron-limited conditions. The study of iron metabolism reveals novel surface molecules that could function as accessible targets for drugs to block iron uptake and, consequently, inhibit pathogen's proliferation.

## Introduction

Iron is the most common cofactor in biology. This fact could be explained by the high abundance of the element in nature and by its chemical properties, mainly by its redox ability. Since iron participates in several metabolic pathways, either aerobes or anaerobes must obtain ferrous iron (Fe^2+^), since ferric iron (Fe^3+^) is insoluble. Typically, fungi accumulate iron through two different strategies: (1) receptor-mediated internalization of ferric-siderophore complexes and/or heme group; and (2) reductive iron assimilation (RIA), involving iron reduction, followed by a ferroxidase-permeation step (Kosman, 2013). The first strategy has been recently described in *Paracoccidioides* spp. It has been shown that this fungus is able to utilize siderophores and hemoglobin as iron sources through receptor-mediated pathways (Bailão et al., 2014; Silva-Bailão et al., 2014). However, the RIA in *Paracoccidioides* spp. remains elusive.

Ferric reductases have a central role in both strategies of iron capture (Baek et al., 2008). These enzymes, along with ferroxidases and permeases, promote high-affinity iron uptake (Kosman, 2003). Moreover, ferric reductases are important in removing iron from siderophores (Yun et al., 2001) or from host iron sources, such as transferrin and hemin (Knight et al., 2005; Saikia et al., 2014). In addition to the essential role in iron uptake, ferric reductases are also important for melanin production, resistance to azole drugs, virulence (Saikia et al., 2014) and host adaptation (Hu et al., 2014) in *Cryptococcus neoformans*. In *C. albicans*, the ferric reductases also participate in the oxidative stress response, filamentous development and virulence (Xu et al., 2014). Besides the transmembrane ferric reductase enzymes, a secreted γ-glutamyltransferase (Ggt1) activity has been described in *Histoplasma capsulatum, Blastomyces dermatitidis, Paracoccidioides* sp. and *Sporothrix schenckii* (Zarnowski and Woods, 2005).

Multicopper oxidases (MCOs) consist of four enzyme superfamilies: laccases, ascorbate oxidases, ferroxidases, and ceruloplasmin, that catalyze the four-electron reduction of molecular oxygen to two molecules of water (Sirim et al., 2011). In fungal ferroxidases, as noted for *Saccharomyces cerevisiae* Fet3, the specificity for Fe^2+^ as an electron donor derives from the presence of two carboxyl groups, E185 and D409, which are in H-bond contact with the T1 Cu ligands H489 and H413, respectively (Kosman, 2010). In most fungi, the Fe^3+^ product derived from a ferroxidase reaction is channeled to a high-affinity iron permease, Ftr1, which is associated to the ferroxidase in the fungal plasma membrane (Kwok et al., 2006; Ziegler et al., 2011).

The *Paracoccidioides* genus comprises thermodimorphic fungal pathogens causing paracoccidioidomycosis (PCM), which is a deep systemic mycosis with a high prevalence in Brazil, Colombia, Venezuela, and Argentina (San-Blas et al., 2002). Since the notification of PCM cases is not obligatory, prevalence of this fungal disease is likely significantly under-reported (Martinez, 2010). The treatment available for PCM is long and may include several side effects and pathogen resistance. Thus, there is a great demand for the development of safer alternative therapies that are able to overcome resistance (Bocca et al., 2013). In this context, blocking iron acquisition is a good strategy to prevent or treat fungal diseases since iron is an essential nutrient for pathogen proliferation in vertebrate hosts (Kronstad et al., 2013). In this way, the knowledge about *Paracoccidioides* spp. iron acquisition mechanisms can contribute to reveal new targets to antifungal therapy.

The iron uptake mechanisms in *Paracoccidioides* spp. have been investigated in part (Parente et al., 2011; Silva et al., 2011; Bailão et al., 2014; Silva-Bailão et al., 2014). It has been demonstrated by our group that *Paracoccidioides* spp. presents multiple ferric reductases and MCOs and a *ggt1* homolog, but no *ftr1* homolog (Silva et al., 2011). Moreover, it was observed that ferric reductase transcripts (Bailão et al., 2006, 2007, 2012a), zinc regulated transporters (Zrts) homologs (Bailão et al., 2006), hemoglobin receptor homologs and transcripts related to siderophore synthesis and uptake (Parente et al., 2011; Silva-Bailão et al., 2014) are induced during *in vitro* iron deprivation or when *in vivo* models of infection were used. Siderophore production and acquisition and host iron sources have been investigated in *Paracoccidioides* spp. In iron-depleted condition, siderophore secretion by this fungus increases. Moreover, *Paracoccidioides* spp. are able to use siderophores as iron source, increasing the fungus ability to survive inside macrophages, a poor-iron environment (Silva-Bailão et al., 2014). We also demonstrated that hemoglobin is the preferential host iron source for *Paracoccidioides* spp. To acquire hemoglobin, the fungus presents hemolytic activity and the ability to internalize the entire molecule instead of promoting the iron release extracellularly. A GPI-anchored hemoglobin receptor, Rbt5, is a virulence factor (Bailão et al., 2014). Since *Paracoccidioides* spp. do not express an Ftr1 homolog, Fre homologs could reduce the iron, which could be imported by iron/zinc permeases (Bailão et al., 2007; Silva et al., 2011), but this hypothesis remains elusive.

In this work, the RIA pathway was investigated. We have demonstrated that *Paracoccidioides* spp. is able to reduce iron, and in the case of *Pb*18, this reductase activity is linked to ferric iron uptake. In contrast, this reductase activity in *Pb*01 does not appear to be up-stream from this uptake. After reduction, the data suggest that Fe^2+^ is probably internalized through a Fe/Zn permease (Zrt). This suggestion is because *Paracoccidioides* spp. genomes do not present an *ftr1* homolog and the *zrt1* and *zrt2* transcripts are up-regulated during iron deprivation. In addition, transcripts related to siderophore uptake and biosynthesis are up-regulated upon iron deprivation. The data suggest that the fungus could use both a non-classical RIA, comprising ferric reductases and Fe/Zn permeases, and siderophore uptake pathways under iron-limited conditions.

## Materials and methods

### Strains and growth conditions

*Paracoccidioides Pb*01 (ATCC MYA-826; *Paracoccidioides lutzii*) (Teixeira et al., 2014) and *Pb*18 (ATCC 32069; *Paracoccidioides brasiliensis*, phylogenetic species S1) (Carrero et al., 2008) were used in this work. The fungus was maintained in brain heart infusion (BHI) medium supplemented with 4% (w/v) glucose at 36°C to cultivate the yeast form. Before each experiment, the cells were grown in liquid BHI supplemented with 4% (w/v) glucose for 72 h at 36°C under rotation.

### *In silico* sequences analysis

*Pb*01 and *Pb*18 putative ferric reductase and ferroxidase amino acid sequences were obtained in the *Paracoccidioides* genome database (http://www.broadinstitute.org/annotation/genome/paracoccidioides_brasiliensis/MultiHome.html). The *Paracoccidioides* spp. sequences were compared using the ClustalX2 program (Larkin et al., 2007). Comparisons were performed with amino acid sequences from other fungi, as following: *Aspergillus fumigatus* (http://www.aspergillusgenome.org), *Aspergillus nidulans* (http://www.broadinstitute.org/annotation/fungi/aspergillus_nidulans_old), *H. capsulatum* (http://www.broadinstitute.org/annotation/genome/histoplasma_capsulatum/MultiHome.html). *Coccidioides immitis, Coccidioides posadasii* (http://www.broadinstitute.org/annotation/genome/coccidioides_group/MultiHome.html), *B. dermatitidis* (http://www.broadinstitute.org/annotation/genome/blastomyces_dermatitidis/MultiHome.html), *Ustilago maydis* (http://www.broadinstitute.org/annotation/genome/ustilago_maydis/Home.html), *C. neoformans* (http://www.broadinstitute.org/annotation/genome/cryptococcus_neoformans/MultiHome.html), *S. pombe* (http://www.pombase.org), *C. albicans* (http://www.candidagenome.org/), and *S. cerevisiae* (http://www.yeastgenome.org). ClustalX2 program (Larkin et al., 2007) and TreeView v.1.6.6 program (Page, 1996) were used for phylogenetic analysis and visualization, respectively, applying the neighbor-joining method and the tree architecture was inferred from 1000 bootstraps. Domains in amino acid sequences were localized using SMART online tool (http://smart.embl-heidelberg.de/).

A structural model of the putative ferroxidase, *Pb*01 PAAG_06004 was obtained using the Modeler program (Sali et al., 1995) at the Max-Planck Institute for Developmental Biology website (http://toolkit.tuebingen.mpg.de/modeller#). The input for the model used the structure of *S. cerevisiae* Fet3 as template (PDB 1ZPU). InsightII was used subsequently to further energy minimize the model using the cvff forcefield with 5000 iterations and a CG convergence of 1.0.

### RNA extraction and quantitative real time PCR (qRT-PCR)

*Pb*01 and *Pb*18 yeast cells were incubated in Synthetic Complete medium (SC medium: 6.67 g/l yeast nitrogen base without amino acids, 2% glucose plus amino acids mixture) with no supplementation or with addition of 200 μM of bathophenanthroline disulfonic acid (BPS) or 10 μM FeCl_3_ at 36°C under rotation. After 2 h, the cells were harvested and total RNA was extracted using TRIzol (TRI Reagent, Sigma-Aldrich, St. Louis, MO, USA) and mechanical cell rupture (Mini-Beadbeater—Biospec Products Inc., Bartlesville, OK). The total RNA was treated with DNAse I (Promega Corporation, Madison, WI, USA) and used as template in *in vitro* reverse transcription (SuperScript III First-Strand Synthesis SuperMix; Invitrogen, Life Technologies). Then, the cDNAs were submitted to a qRT-PCR reaction, which was performed using SYBR Green PCR Master Mix (Applied Biosystems, Foster City, CA) in a StepOnePlus Real-Time PCR System (Applied Biosystems Inc.). The expression values were calculated using the transcript that encoded alpha tubulin (XM_002796593) as the endogenous control as previously reported (Bailão et al., 2012b). The annealing temperature for all primers was 62°C. The qRT-PCR reaction was performed in biological triplicate for each cDNA sample, and a melting curve analysis was performed to confirm single PCR products. The relative standard curve was generated using a pool of cDNAs from all the conditions that were used, which was serially diluted 1:5–1:625. Relative expression levels of transcripts of interest were calculated using the standard curve method for relative quantification (Bookout et al., 2006). Student's *t*-test was applied in the statistical analyses. For confirmation of Zrts induction in iron-deprived condition by qRT-PCR, all steps were performed as described, except for *Pb*01 incubation in chemically defined MMcM medium (Restrepo and Jiménez, 1980) supplemented with 3.5 μM inorganic iron [Fe(NH_4_)_2_(SO_4_)_2_] or with 50 μM of the iron chelator bathophenanthroline disulfonic acid (BPS: Sigma-Aldrich, Germany) for 3 or 24 h before cell harvesting.

### TTC indicator plates

To detect *Pb*01 and *Pb*18 surface reductase activity, a plate assay was used as described previously (Ogur et al., 1957), except that the 2,3,5-triphenyltetrazolium chloride (TTC)-containing overlay was poured on fresh colonies grown on SC plates, supplemented or not with 50 μM BPS (no iron condition), 30 μM inorganic iron [Fe(NH_4_)_2_(SO_4_)_2_], 30 μM hemoglobin, 120 μM hemin, 30 μg/ml ferritin, or 30 μM transferrin, independently. All host iron sources were purchased from Sigma-Aldrich, St. Louis, MO, USA.

### ^59^Fe uptake assays

After 24 h of *Pb*01 and *Pb*18 incubation in SC medium with no supplementation or with addition of 200 μM BPS or 10 μM FeCl_3_ at 36°C under rotation, the cells were harvested and washed with 1 mM EDTA in citrate uptake buffer (2% analar glucose, 0.1 MES buffer, and 20 mM Na-citrate, pH 6.0). After, the cells were washed twice with citrate uptake buffer and incubated for 15 min at 36°C under rotation. At this time, an aliquot of cells was collected and counted in a hemocytometer. Then, 20 mM of ascorbic acid was added (reductive-independent ^59^Fe uptake assay) or not (reductive-dependent ^59^Fe uptake assay); Cl_4_K_2_Pt (PtII compound) was added or not; and 0.2 μM ^59^Fe solution (Perkin-Elmer, Waltham, MA) was added independent of the condition. A triplicate of point 0 and 60 min were collected in a filter membrane (type A/C 25 mm glass fiber filter, Sigma-Aldrich, St. Louis, MO, USA) and the cells were washed with ice-cold 1X Quench buffer (37.5 mM succinic acid, 62.5 mM Tris, and 12.8 mM EDTA, pH 6.0). Then filters containing the cells were placed on the bottom of the test tubes and internalized ^59^Fe was measured using a Wallac γ counter (LKB Wallac CompuGamma). ^59^Fe values were normalized with the number of cells.

### High-throughput mRNA sequencing (RNA-seq)

*Pb*01 yeast cells were incubated in chemically defined MMcM medium supplemented with 3.5 μM Fe(NH_4_)_2_(SO_4_)_2_ or with 50 μM BPS (Sigma-Aldrich, Germany) for 24 h at 36°C on a rotary shaker at 150 rpm. After that, the cells for both conditions, in biological triplicates, were treated with TRIzol reagent (Invitrogen, Carlsbad, CA, USA) to obtain RNA molecules. The cDNAs libraries were prepared from poly(A)-fragment selected mRNA and processed on the Illumina HiSeq2000 Sequencing System (http://www.illumina.com/). As a result, approximately 40 million of reads of 100 bp paired-end sequencing were obtained for each sample. The sequencing reads were mapped to reference the *Pb*01 genome (http://www.broadinstitute.org/annotation/genome/paracoccidioides_brasiliensis/ MultiHome.html) using the Bowtie 2 tool (Langmead et al., 2009). Briefly, each read was allowed to alignment in just one site of the genome and the reads were counted. The default parameters were used to perform the alignment. The number of mismatches allowed in seed alignment (−N) is 0, and the length of each seed (−L) is 20. Reads mapped to a gene region were counted to infer expression measurement. Differentially expressed genes were identified using Fisher Exact test in DEGseq package (Wang et al., 2010). A *p*-value lower than 0.001 and minimum fold change of 1.5 were used to filter the most relevant candidates. Meaning that transcripts with log2 (fold change) higher than 0.58 or less than −0.58 were selected and classified as up- and down-regulated transcripts, respectively. Transcript's identifications and annotations were determined from the *Paracoccidioides* genome database. The biological processes were obtained using the Pedant on MIPS (http://pedant.helmholtzmuenchen.de/pedant3htmlview/pedant3view?Method=analysis&Db=p3_r48325_Par_brasi_Pb01) which provides a tool to browse and search the Functional Categories (FunCat) of proteins.

The predicted proteins encoded by the transcripts identified through RNAseq strategy were also classified as copper−, iron−, and zinc-binding proteins from *Paracoccidioides* genome as previously described (Tristão et al., 2015). Briefly, metalloproteins were identified by using the RDGB tool (Andreini et al., 2011) with default options. In the RDGB strategy, the protein domains defined in the Pfam library are used to identify putative homologs in any desired genome or list of genomes. Copper−, iron−, and zinc-binding Pfam domains were initially identified in the sequence of copper−, iron−, and zinc-binding proteins of known 3D structures, which are available from the Protein Data Bank (PDB). When a particular metal is present within the 3D structure of the protein, this information can be readily extracted from the PDB database along with the pattern of amino acids that are involved in the interaction of the protein with the metal. In addition, other specific domains were also identified to predicted encoded transcripts using the *Paracoccidioides* genome database (http://www.broadinstitute.org/annotation/genome/paracoccidioides_brasiliensis/MultiHome.html).

## Results

### Characterization of *Paracoccidioides* spp. ferric reductases

The *Paracoccidioides* spp. Genome Database searching for proteins possessing all the three domains: FRD (PF01794), FAD-binding domain (PF08022), and ferric reductase NAD-binding domain (PF08030) resulted in seven *Pb*01 and seven *Pb*18 putative ferric reductases. Moreover, amino acid sequences of *Paracoccidioides* spp. Ggt1 homologs were collected in the same database. These sequences were used to construct a phylogenetic tree comprising ferric reductase sequences of different fungi: *A. nidulans, A. fumigatus, H. capsulatum, C. immitis, C. posadasii, B. dermatitidis, U. maydis, C. neoformans, S. pombe, S. cerevisiae*, and *C. albicans* (Supplementary Figure 1). It could be observed that all the *Paracoccidioides* spp. sequences are grouped with *H. capsulatum, C. immitis, C. posadasii*, and *B. dermatitidis* sequences. Moreover, BLAST analyses revealed the presence of three groups of proteins in this phylogenetic three: (1) transmembrane ferric reductases; (2) NADPH oxidases; and (3) γ-glutamyltransferases. The sequences identified as NADPH oxidases were excluded from posterior analyses, since they are out of the manuscript's scope.

**Figure 1:**
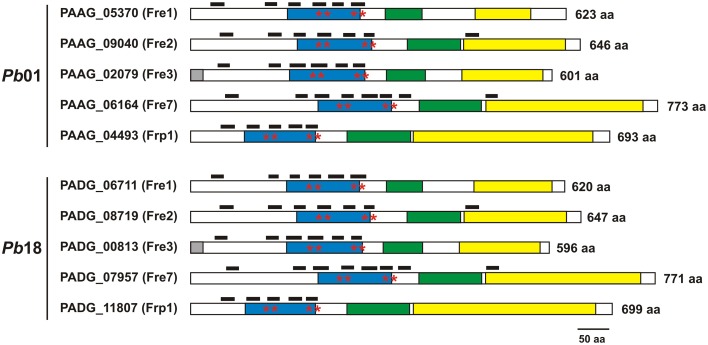
***Paracoccidioides* spp. ferric reductases present typical domains**. Domains identified in *Pb*01 and *Pb*18 transmembrane ferric reductases were: ferric reductase domain (blue box), FAD-binding domain (green box), and NAD-binding domain (yellow box). Asterisks indicate the bis-hememotif comprising conserved histidine residues. Black boxes above each sequence indicate the lengh and position of transmembrane domains. The number of amino acids (aa) of each protein is indicated adjacent to the sequences.

All the *Pb*01 and *Pb*18 sequences classified as transmembrane ferric reductases contained characteristic features of ferric reductases, such as the FRD, NAD, and FAD-binding domains, a bis-heme motif and at least five transmembrane domains (Figure 1). The sequences classified as γ-glutamyltransferases were aligned and presented similarity, mainly in some regions of γ-glutamyltranspeptidase domain (PF01019) (Supplementary Figure 2), indicating that other fungi, such as *A. fumigatus, A. nidulans, C. albicans, C. neoformans, S. cerevisiae, S. pombe, U. maydis, C. immitis*, and *C. posadasii*, not tested yet for extracellular glutathione-dependent ferric reductase (GSH-FeR) activity could utilize this alternative iron reduction route.

### *Paracoccidioides* spp. ferric reductase regulation and iron uptake

**Figure 2:**
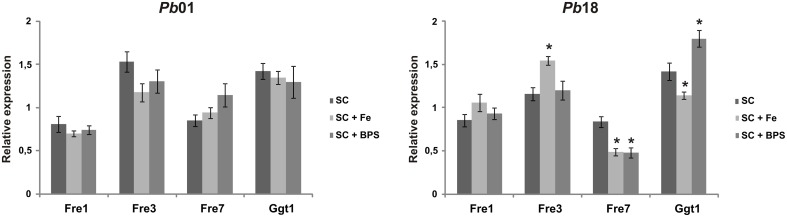
**Expression profile of transcripts putatively encoding *Paracoccidioides* spp. ferric reductases**. *Pb*01 and *Pb*18 yeast cells were recovered from SC medium, which was supplemented or not with 200 μM BPS or 10 μM FeCl_3_, after 2 h incubation. After RNA extraction and cDNA synthesis, *Pb*01 and *Pb*18 transcript levels were quantified using qRT-PCR. The expression values were calculated using alpha tubulin as the endogenous control. The data are expressed as the mean ± SD from triplicates. ^*^Statistically significant data as determined by Student's *t*-test (*p* < 0.05).

In order to evaluate the *Pb*01 and *Pb*18 putative ferric reductase transcripts, the yeast cells were cultivated in presence of 10 μM inorganic iron or 200 μM BPS. After 2 h, it was observed no relevant regulation of all *Pb*01 transcripts analyzed in response to iron availability. *Pb*18 *fre3, fre7*, and *ggt1* transcripts presented a slight regulation in response to iron availability. Under iron supplementation, *Pb*18 *fre3* transcript level increased, whereas *Pb*18 *fre7* and *ggt1* transcripts level reduced, comparing to the SC medium without any supplementation. Under iron deprivation (presence of BPS), *Pb*18 *fre7* transcript level reduced, whereas *Pb*18 *ggt1* transcript level increased, comparing to the SC medium without any supplementation (Figure 2). Overall, there was no indication of a pattern of iron regulation of ferric reductase transcripts abundance in *Paracoccidioides* spp.

**Figure 3:**
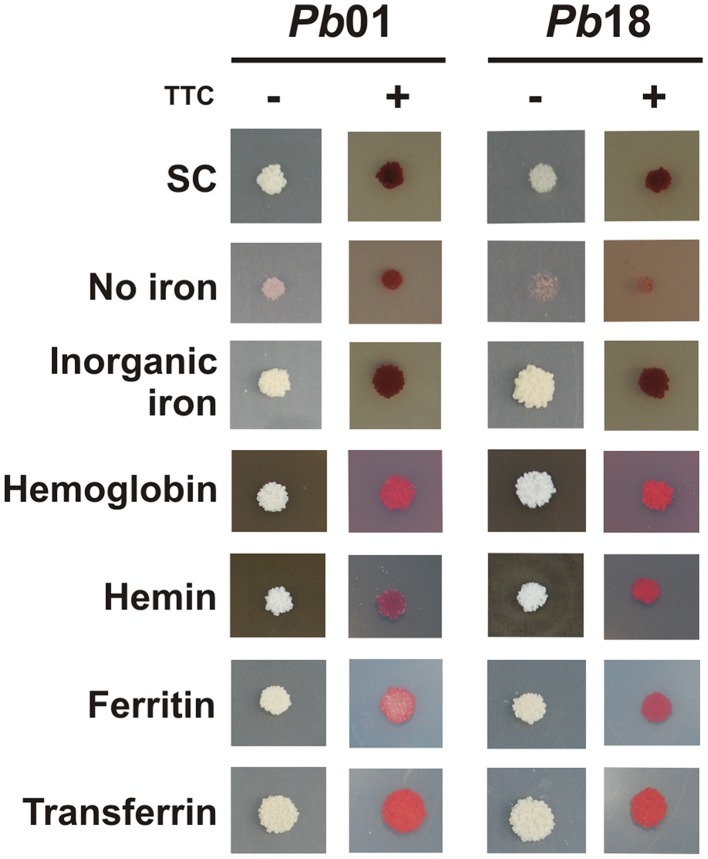
**Activation of ferric reductase activity in presence of different iron sources by *Paracoccidioides* spp**. *Pb*01 and *Pb*18 cell cultures were collected after 24 h in presence of 200 μM BPS, washed and 10^4^ cells were spotted on SC medium plates, which were supplemented or not with 50 μM BPS, 30 μM inorganic iron, 30 μM hemoglobin, 120 μM hemin, 30 mg/ml ferritin, or 30 μM transferrin, independently. After 10 days of growth, TTC-containing agar solution was poured on the plates containing the colonies. The red colonies after TTC addition indicate a reduction of the colorless electron acceptor TTC to a red formazan precipitate.

**Figure 4:**
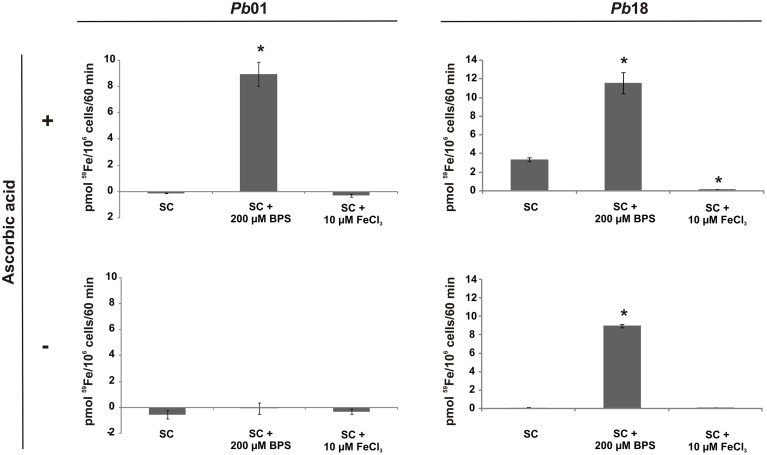
**The iron uptake rate in *Paracoccidioides* spp. is dependent on the culture conditions and on the oxidation state of available iron**. *Pb*01 and *Pb*18 yeast cells were cultivated on SC medium, which was supplemented or not with 200 μM BPS or 10 μM FeCl_3_, for 24 h. Then, the cells were collected, washed and incubated in presence of ^59^Fe with addition (+) or not (−) of ascorbic acid for 60 min. After this, the cells were collected and washed again and submitted to a γ counter for iron uptake rate establishment. The data is present as the mean ± SD from triplicates or quadruplicates. ^*^Statistically significant data, as determined by Student's *t*-test (*p* < 0.05), relative to yeast cells cultivated on SC medium.

To investigate if the RIA pathway could be functional when *Paracoccidioides* spp. grow in presence of different iron sources, inorganic iron, hemoglobin, hemin, ferritin, or transferrin were used. SC medium not supplemented with iron or chelated with BPS (no iron) were also used as controls. The *Pb*01 and *Pb*18 yeast cells growing in all conditions presented cell surface reductase activity, as evidenced by the red colony color in the presence of TTC (Figure 3). In addition, ^59^Fe uptake was quantified in presence or absence of the reducing agent ascorbic acid using *Pb*01 and *Pb*18 yeast cells grown in presence or absence of iron. The results showed that cells cultivated under iron deprivation (200 μM BPS) presented an increased ^59^Fe uptake rate, when compared to cells cultivated in the presence of 10 μM FeCl3 (Figure 4). When ascorbic acid was omitted, ^59^Fe uptake should be dependent on ferric reduction; only *Pb*18 cells cultivated under iron deprivation were able to internalize ^59^Fe added as ferric iron indicating that the reductase activity in this strain was coupled to iron uptake.

**Figure 5:**
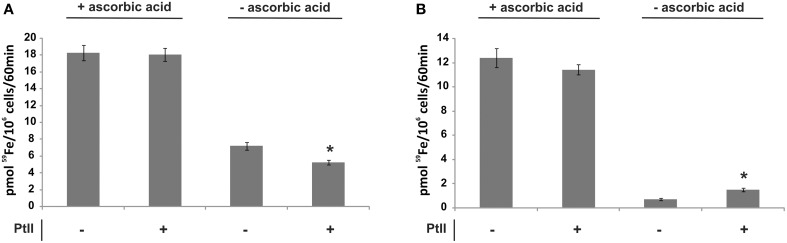
**Effect of a platinum compound addition on the iron uptake rate of *Paracoccidioides* spp**. *Pb*01 and *Pb*18 yeast cells were cultivated on SC medium supplemented or not with 200 μM BPS for 24 h. Then, the cells were collected, washed and incubated in presence of ^59^Fe with addition (+) or not (−) of ascorbic acid and/or a platinum compound (PtII) for 60 min. After this, the cells were collected and washed again and submitted to a γ counter for iron uptake rate establishment in *Pb*18 **(A)** and *Pb*01 **(B)**. The data is present as the mean ± SD from triplicates or quadruplicates. ^*^Statistically significant data, as determined by Student's *t*-test (*p* < 0.05), relative to yeast cells cultivated on abscence of a PtII compound.

Since the ferric reductase activity can be subject to inhibition by Pt(II) (Eide et al., 1992), the *Pb*18 ferric reductase sensitivity to this element was tested using as an indicator the sensitivity of ^59^Fe uptake to this transition metal. *Pb*18 cells cultivated under iron deprivation exhibited a modest decrease in reductase-dependent ^59^Fe uptake in the presence of Pt(II) (Figure 5A); the limited uptake by *Pb*01 of ^59^Fe added as ferric iron was not strongly affected by this addition (Figure 5B).

### The multicopper oxidases in *Paracoccidioides* spp.

To start an investigation about *Paracoccidioides* spp. ferroxidases, a searching for MCOs in Laccase Engineering Database (LccED: http://www.lcced.uni-stuttgart.de) was developed. It resulted in no *Paracoccidioides* spp. sequences classified in family E, corresponding to fungal ferroxidases. *Paracoccidioides* spp. MCOs are classified as basidiomycete laccases (family A), ascomycete laccases (family B), and fungal pigment MCOs (family D) (http://www.lcced.uni-stuttgart.de/cgi-bin/LccED1.2/index.pl?page=org&id=35). However, the sequences of all the *Pb*01 and *Pb*18 MCOs deposited in *Paracoccidioides* Genome Database were collected and aligned with *S. cerevisiae* Fet3 (Supplementary Figure 3) to identify both potential Cu-binding residues and the carboxylate side chains that support ferrous oxidase activity in fungal ferroxidases (Kosman, 2010). This examination indicated that only PAAG_00163 do not clearly displayed all four Cu-binding motifs and therefore cannot support the ferroxidase activity typical in the RIA pathway.

**Figure 6:**
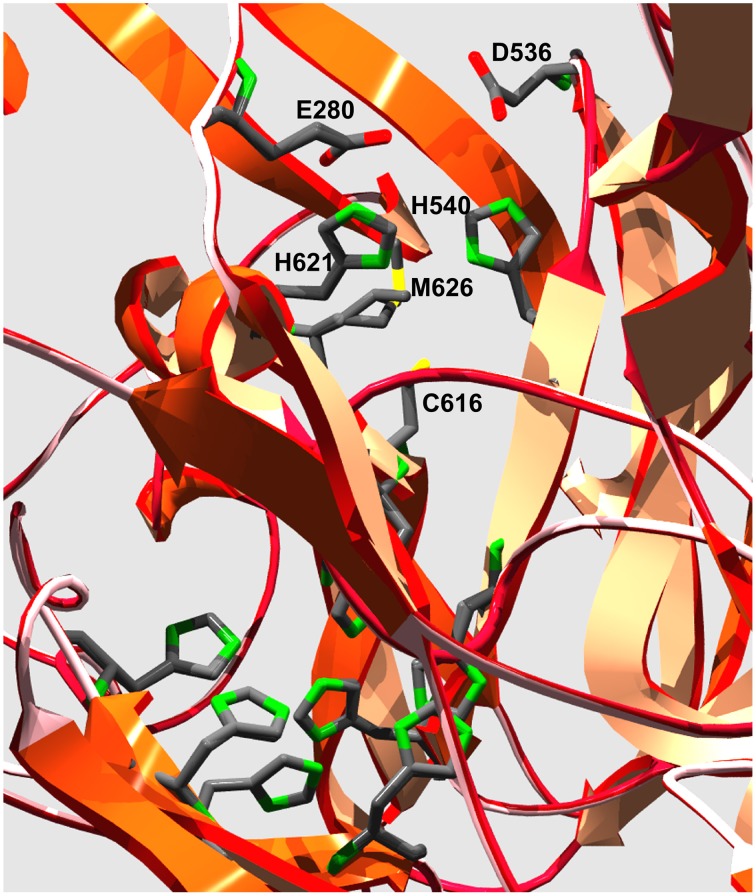
**Model of the putative Cu-coordination sites in *Pb*01 PAAG_06004**. The ribbon diagram illustrates the protein folds that contain the 11 side chains that make up the coordination sites for the 4 Cu-atoms found in fungal MCOs. The six His side chains in the lower left quadrant are ligands to the T2 and T3 Cu atoms that make up the trinuclear cluster to which O_2_ binds and is reduced to 2H_2_O. The grouping of H540, C616, H621, and M626 are ligands to the T1 Cu that is the electron acceptor from the reducing substrate. Ferroxidases contain two acidic side chains outer-sphere to this ligand grouping that are in H-bond contact with each of the His side chains. The unbiased computer model of PAAG_06004 has E280 and D536 in this conformation relative to the Fet3 T1 Cu His ligands H621 and H540, respectively, suggesting that this protein could exhibit ferroxidase activity.

The ferroxidase activity in fungal MCOs is due to two acidic residues; in Fet3 these are E185 and D407. The global alignments (Supplementary Figure 3) provide insight into the presence of homologous residues in two *Paracoccidioides* spp. proteins, PAAG_06004 and PADG_05994, which are likely ferroxidases. A clear difference between Fet3 and *Paracoccidioides* spp. proteins is that the former presents a C-terminal transmembrane domain, whereas PAAG_06004 and PADG_05994 present N-terminal transmembrane domain (Supplementary Figure 3). Due to high similarity in ferroxidase motifs and in residues involved in Fe^2+^ binding between the PAAG_06004 and PADG_05994, a structural model of PAAG_06004 was constructed based on the structure of Fet3 (PBD 1ZPU). Despite the fact that the two proteins have only 22% identity, a satisfactory model was generated that did, however, contain a relatively large (unfavorable) Z-score for torsion angle energy. However, the model faithfully reproduced the conformation of the protein's four predicted Cu-coordination sites and indicated that in PAAG_06004 E280 and D536 could be part of a ferroxidase site in this protein (Figure 6). Isolation and characterization of this protein is necessary to test this possible ferroxidase activity.

### The iron permeases in *Paracoccidioides* sp.

A whole transcriptome sequencing strategy was performed as previously described (Lima et al., 2014). The number of the reads counted for each transcript in iron replete and iron-deprived conditions was represented by scattered dots (Supplementary Figure 4). The transcripts were represented by dots, which could present a different number of reads in each condition (Supplementary Figure 4A). We also applied a statistical test to identify differentially expressed transcripts, represented by red dots (Supplementary Figure 4B). A total of 549 transcripts were statistically significant (Supplementary Figure 4B) but a cut-off of 1.5-fold change (Amich et al., 2013) generated 30 up- and 44 down-regulated transcripts (Supplementary Tables 1, 2).

**Figure 7:**
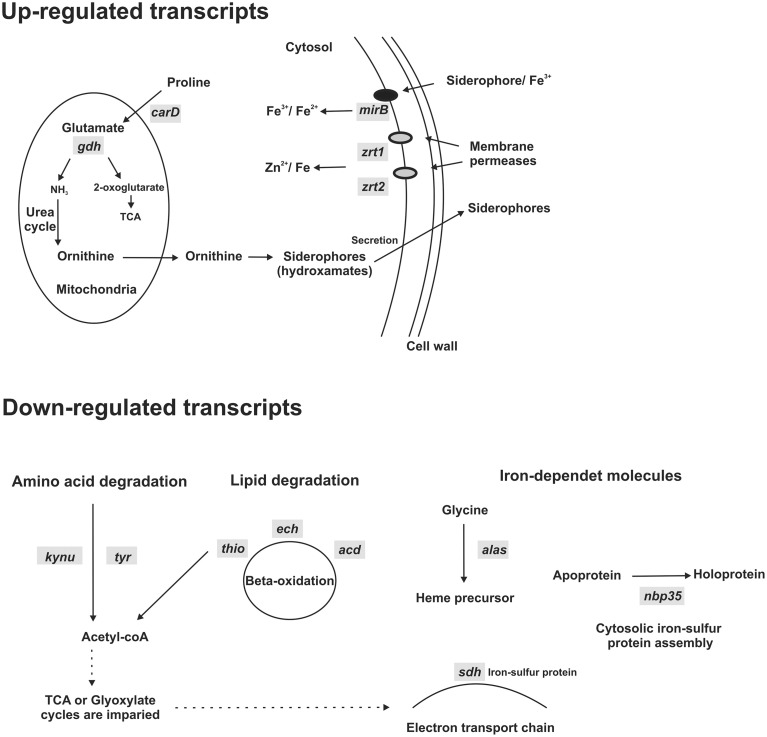
**Schematic representation of up and down-regulated transcripts under iron deprivation**. *Pb*01 yeast cells were cultivated on MMcM medium supplemented with 3.5 μM inorganic iron or with 50 μM BPS (iron deprivation) for 24 h. Then, a high-throughput mRNA sequencing (RNA-seq) strategy was used to compare transcripts expressed in iron deprivation and in presence of iron. Some transcripts obtained using this strategy was selected to construct this scheme. In iron deprivation condition, transcripts involved with siderophore synthesis (*carD* and *gdh*) and uptake (*mirB*) and with iron uptake using Fe/Zn permeases (*zrt1* and *zrt2*) were up-regulated. On the other hand, transcripts involved with amino acid (*kynu* and *tyr*) and lipid degradation (*thio, ech*and *acd*) and with synthesis of putative iron-dependent molecules (*alas, sdh*, and *nbp35*), such as heme and iron-sulfur proteins, were down-regulated. *carD*, carbapenemantibiotics biosynthesis protein; *gdh*, NADP-specific glutamate dehydrogenase; *mirB*, siderophore iron transporter; *zrt1* and *zrt2*, zinc regulated transporters; *kynu*, kynureninase; *tyr*, tyrosinase central domain-containing protein; *thio*, 3-ketoacyl-CoA thiolase; *ech*, delta(3,5)-delta(2,4)-dienoyl-CoA isomerase; *acd*, acyl-CoA dehydrogenase family protein; *alas*, 5-aminolevulinate synthase; *sdh*, succinate dehydrogenase iron-sulfur subunit; *nbp35*, cytosolic Fe-S cluster assembling factor.

Previous analysis has indicated that *Paracoccidioides* spp. does not express a Ftr1 homolog and thus the fungus could use zinc permeases to transport iron (Bailão et al., 2007; Silva et al., 2011). The RNAseq analysis revealed that zinc permeases transcripts (*zrt1* and *zrt2*) are up-regulated under iron deprivation in *Pb*01 yeast cells, consistent with this hypothesis (Supplementary Table 1 and Figure 7). The results also revealed that a siderophore uptake encoding transcript (*mirB*) and transcripts encoding enzymes, such as carbapenemantibiotics biosynthesis protein (*carD*) and NADP-specific glutamate dehydrogenase (*gdh*), involved in ornithine biosynthesis, a hydroxamate precursor, are up-regulated under iron deprivation (Supplementary Table 1 and Figure 7). This fact points to an increase in siderophore synthesis and uptake during iron deprivation. Down-regulated transcripts are related to amino acid and lipid metabolism, which could generate acetyl-CoA, precursor for several metabolic pathways, and with heme biosynthesis, an iron-dependent molecule. The acetyl-CoA decreased production in association with the down-regulation of the electron transport chain could indicate an impaired aerobic metabolism (Figure 7). In fact, iron-sulfur proteins, such as succinate dehydrogenase iron-sulfur subunit (*sdh*) and cytosolic Fe-S cluster assembling factor NBP35, that also need iron as a cofactor, were down-regulated (Supplementary Table 2 and Figure 7), indicating that the fungus faces an iron-deprived condition.

**Figure 8:**
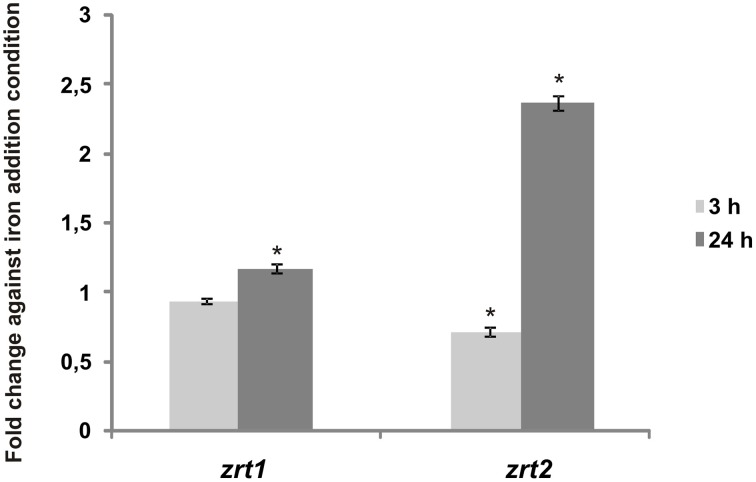
**Transcripts putatively encoding *Paracoccidioides* sp. Fe/Zn permeases are iron-regulated**. *Pb*01 yeast cells were recovered from MMcM medium, which was supplemented with 50 μM BPS or 3.5 μM inorganic iron for 3 or 24 h. After RNA extraction and cDNA synthesis, levels of *Pb*01 *zrt1* and *zrt2* were quantified by qRT-PCR. The expression values were calculated using alpha tubulin as the endogenous control. The values that were plotted on the bar graph were normalized against the expression data that were obtained from the iron addition condition (fold change). The data are expressed as the mean ± SD from triplicates. ^*^Statistically significant data as determined by Student's *t*-test (*p* < 0.05).

When *Pb*01 *zrt1* and *zrt2* transcripts were analyzed using qRT-PCR, similar results were obtained. After 24 h of iron deprivation, *zrt1* and *zrt2* expression increased (Figure 8), indicating that these genes could play a role in iron acquisition.

### The iron uptake strategies in *Paracoccidioides* spp.

**Figure 9:**
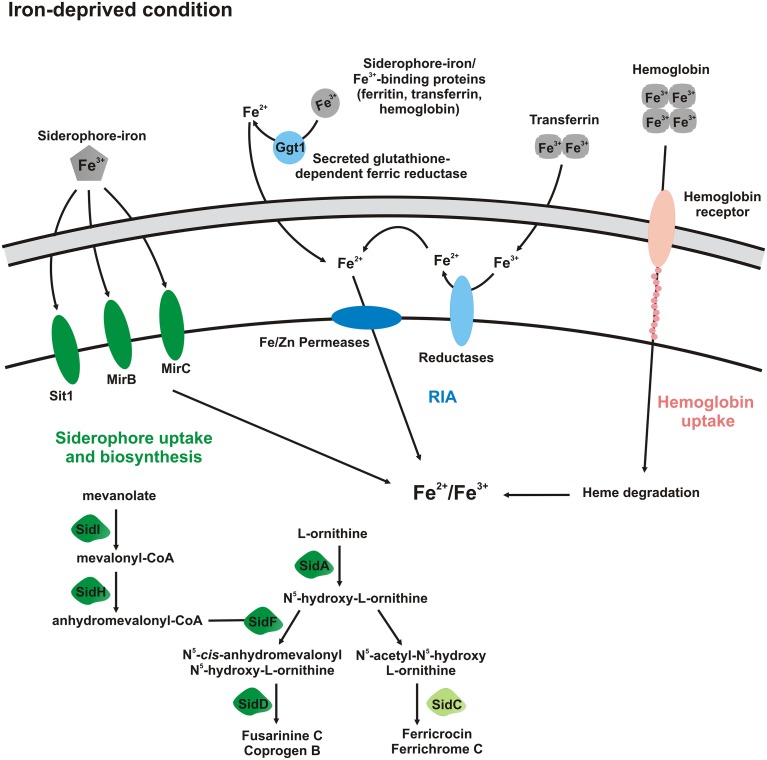
**Schematic representation of *Paracoccidioides* spp. activated pathways in iron-deprived condition**. Forms with dark colors indicate proteins whose transcription is up-regulated and that ones with light colors do not present transcription regulation. In that way, in iron-deprived condition, siderophore uptake, and biosynthesis of extracellular siderophores (coprogen B), as well as, Fe/Zn permeases (Zrts) are up-regulated. It seems that transmembrane ferric reductases and hemoglobin receptors are up-regulated mainly in presence of host iron sources, such as transferrin and hemoglobin, respectively.

Based on previously published data (Bailão et al., 2007, 2014; Parente et al., 2011; Silva-Bailão et al., 2014) and those obtained in this study, an updated model regarding the iron uptake strategies used by *Paracoccidioides* spp. was proposed (Figure 9). Under iron deprivation, the fungus may use both ferric/ferrous iron uptake and siderophore uptake pathways, since an increase in *zrts* transcripts and in those involved with siderophore production and uptake was observed in this study and/or in previously published data (Parente et al., 2011; Silva-Bailão et al., 2014). Hemoglobin uptake through the hemoglobin receptors may be used preferentially in the presence of this host molecule, since *Pb*01 putative hemoglobin receptors present reduced transcription under iron depletion (Bailão et al., 2014). In the same way, transmembrane ferric reductases seem to be up-regulated in presence of host iron sources, such as transferrin and hemoglobin, since *Pb*01 *fre3* transcript (named previously as *fre2*) presented an increased expression during *Pb*01 yeast cells incubation with human plasma (Bailão et al., 2007) and human blood (Bailão et al., 2006).

## Discussion

As described previously, *Paracoccidioides* spp. presents multiple ferric reductases (Silva et al., 2011). These proteins are very similar to homologs found in *H. capsulatum, C. immitis, C. posadasii*, and *B. dermatitidis*, as expected, since all the organisms are classified in the Onygenales order (Sharpton et al., 2009). There are five putative transmembrane ferric reductases either for *Pb*01 or for *Pb*18. Moreover these fungi present one glutathione-dependent ferric reductase homolog, whose activity has been described (Zarnowski and Woods, 2005). The *Paracoccidioides* spp. transmembrane ferric reductases present domains described for homologs from other pathogenic fungi, such as FRD, NAD and FAD-binding domains, bis-heme motif and transmembrane domains (Almeida et al., 2009; Saikia et al., 2014). The expression of these ferric reductases seems not to be iron-dependent, since the differences observed between the iron-replete and the iron-deprived conditions were subtle. Similar results were observed for *C. neoformans*, in which subtle differences in ferric reductases expression were observed in presence or absence of iron (Saikia et al., 2014). However, an increase in *Paracoccidioides* spp. ferric reductase expression has been observed in the presence of human blood and plasma (Bailão et al., 2006, 2007). This apparent inconsistency could be explained if the ferric reductase expression would be activated in the presence of human iron sources, such as transferrin, an iron source that could be used by *Paracoccidioides* spp. (Bailão et al., 2014).

^59^Fe uptake assays demonstrated that *Pb*18 reduce and uptake iron in scarcity of this metal. On the other hand, *Pb*01 express an apparently inefficient RIA system, since ^59^Fe uptake in the absence of ascorbic acid was low in any culture condition used in this work although TTC assay indicates this strain does exhibit cell surface ferric reductase activity. It has been proposed that *A. nidulans* also lack an efficient RIA pathway, because a siderophore-deficient strain is not able to utilize Fe^3+^ (Eisendle et al., 2003). Perhaps the main route to acquire iron in these fungi is the siderophore uptake, which has been demonstrated to be efficient either in *A. nidulans* (Eisendle et al., 2003) or in *Paracoccidioides* spp. (Silva-Bailão et al., 2014).

*Paracoccidioides* spp. MCOs (PAAG_06004 and PADG_05994), with a low Fet3 identity, were identified in *Paracoccidioides* spp. genome database. To confirm if these proteins could act as ferroxidases and if the MCO domain face or not the cytoplasm, the proteins should be isolated, characterized, and cellular localized, that is our focus in future studies. What we hypothesize at this moment is that an MCO does not play a significant role in RIA pathway in *Paracoccidioides* spp., since in previous work (Bailão et al., 2006, 2007, 2014; Costa et al., 2007; Parente et al., 2011), as well as in this work, ferroxidase regulation in low-iron conditions was not observed. Moreover, this pathway appears to be unusual, since it does not include an Ftr1 homolog, common to several fungi (Stearman et al., 1996; Ziegler et al., 2011). Similarly, a homology search in *A. nidulans* genome database failed to identify Fet3 and Ftr1 homologs (Eisendle et al., 2003).

We note, however, that *Paracoccidioides* spp. express Zrts homologs that are induced in iron- (this work) or zinc-deprivation (Parente et al., 2013). In plants and mammals, it has been demonstrated that ZIP (Zrt/Irt-like Proteins) family of membrane transporters is able to transport not only zinc, but also iron (Zhao et al., 2010; Milner et al., 2013). Additionally, it has been observed that *Paracoccidioides* spp. Zrts present more than one possible metal-binding domain (HXHXHXH) (Zhao and Eide, 1996) in the sequence (Silva et al., 2011), suggesting that these domains could be responsible for zinc and iron binding. These facts corroborate the hypothesis that *Paracoccidioides* Zrts are able to transport both iron and zinc. Moreover, it has been demonstrated that Zrt expression increases in yeast cells recovered from liver of infected mice and in presence of human plasma (Bailão et al., 2006, 2007). These data suggest that the permease is regulated in iron-limited conditions either *in vitro* or *in vivo* and in presence of human iron sources, such as transferrin. Maybe the iron-regulation in *Paracoccidioides* spp. impacts most strongly on the permeases and not on the ferric reductases.

A whole transcriptome sequencing strategy also revealed that both a siderophore transporter and the ornithine, a hydroxamate precursor (Eisendle et al., 2003), biosynthesis are up-regulated, indicating that *Paracoccidioides* spp. utilize the siderophore uptake pathway during iron scarcity, as previously demonstrated (Silva-Bailão et al., 2014). At the same time, expression of Zrts is also up-regulated, indicating that the fungus use more than one iron uptake strategy to acquire this metal when in iron scarcity. On the other hand, aerobic respiration and iron-dependent molecule synthesis pathways seems to be down-regulated, such as the synthesis of iron-sulfur proteins and heme precursors. It has been observed that tricarboxylic acid cycle and electron transport chain proteins decreased in abundance under iron limiting conditions (Parente et al., 2011). It could occur because iron is used as prosthetic group in a plenty of energy metabolism pathways (Kosman, 2013).

The RIA pathway in *Paracoccidioides* spp. appears constitutive in that ferric reductase expression is relatively insensitive to type or concentration of iron source. In contrast, in iron-deprived condition (Figure 9), transcripts related to siderophore uptake and biosynthesis of extracellular siderophores (coprogen B) are up-regulated as observed in this work and in others previously published (Parente et al., 2011; Silva-Bailão et al., 2014). Transcripts related to iron and zinc permeases are also up-regulated in *Paracoccidioides* spp. This indicates that the fungus can use both RIA and siderophore uptake pathways in iron limited condition. *Paracoccidioides* spp. also may use hemoglobin receptors to acquire this host iron source; those receptors are activated preferentially in presence of hemoglobin instead of iron deprivation (Bailão et al., 2014). In addition, in presence of transferrin (abundant in human plasma) and hemoglobin (abundant in human blood), the transcription of ferric reductases is up-regulated (Bailão et al., 2006, 2007). These data indicate that both RIA and siderophore uptake pathways could function under iron deprivation.

The study of iron metabolism is important to reveal novel molecules that contribute to iron uptake, an essential micronutrient for almost all organisms (Johnson, 2008). These proteins could function as accessible targets for drugs to block iron uptake, what could impair the pathogen's life. Moreover, iron permeases might also be exploited as vaccine targets or as route for antifungal internalization (Kronstad et al., 2013). Then, *Paracoccidioides* spp. proteins involved with iron uptake could be used as vaccine or drug targets or as drug internalization route to inhibit the proliferation of the pathogen.

### Conflict of interest statement

The authors declare that the research was conducted in the absence of any commercial or financial relationships that could be construed as a potential conflict of interest.
