# Supplementary material for: Paracoccidioides spp. ferrous and ferric iron assimilation pathways
Source: Front Microbiol. 2015 Aug 12;6:821. doi: 10.3389/fmicb.2015.00821 (PMC4585334; doi:10.3389/fmicb.2015.00821)
Supplement: Supplementary file 1 [file Table1.DOCX]

**Supp. Table 1. Some up-regulated transcripts of *Pb*01 yeast cells under iron deprivation for 24 h detected by RNAseq analysis.**

| ID^a^ | Annotation^b^ | Fold change (log2)^c^ | Metal binding/ Specifc domain^d^ | Functional Categories^e^ | | |
| --- | --- | --- | --- | --- | --- | --- |
| METABOLISM | | | | | | |
| Amino acid metabolism | | | | | | |
| PAAG_07689 | NADP-specific glutamate dehydrogenase | 0.71 | No/ Glu,Leu,Phe,Val dehydrogenase, dimerisation domain | [Glutamate biosynthetic process](http://www.ebi.ac.uk/QuickGO/GTerm?id=GO:0006537) | | |
| PAAG_06777 | Carbapenem antibiotics biosynthesis protein carD | 0.77 | No/ Proline dehydrogenase | [Glutamate biosynthetic process](http://www.ebi.ac.uk/QuickGO/GTerm?id=GO:0006537) | | |
| C-compound and carbohydrate metabolism | | | | | | |
| PAAG_05974 | Glyoxalase* | 0.81 | No/ Glyoxalase-like domain | | Aromatic hydrocarbons catabolism | |
| PAAG_06304 | [sterigmatocystin 8-O-methyltransferase *](http://www.ncbi.nlm.nih.gov/blast/Blast.cgi#alnHdr_225556215) | 1.82 | No/ O-methyltransferase. | | C-compound and carbohydrate metabolism | |
| PROTEIN FATE | | | | | |  |
| PAAG_03520 | Arrestin | 0.93 | No/ Arrestin (or S-antigen), N-terminal domain | Protein ubiqutination/ signal transduction | | |
| TRANSPORT | | | | | |  |
| PAAG_01685 | Siderophore iron transporter mirB | 0.90 | No/ Major Facilitator Superfamily and Fungal trichothecene efflux pump (TRI12) | Siderophore-iron transport | | |
| PAAG_08727 | Zinc-regulated transporter 1 | 0.93 | Yes (Zn)/ ZIP Zinc transporter and zip: ZIP zinc/iron transport family | Heavy metal ion transport (Zn, Cu+, Fe^3+^ etc) | | |
| PAAG_03419 | Zinc-regulated transporter 2 | 1.34 | Yes (Zn)/ ZIP Zinc transporter and zip: ZIP zinc/iron transport family | Heavy metal ion transport (Zn, Cu+, Fe^3+^ etc) | | |
| CELL WALL BIOGENESIS | | | | | | |
| PAAG_04235 | Hydrophobin | 0.64 | No/ Fungal hydrophobin | [Structural constituent of cell wall](http://www.ebi.ac.uk/QuickGO/GTerm?id=GO:0005199) | | |
| MISCELLANEOUS | | | | | |  |
| PAAG_00704 | Methyltransferase | 0.58 | No/ Methyltransferase domain | Methyltransferase activity | | |
| PAAG_01677 | FAD-dependent pyridine nucleotide-disulphide oxidoreductase * | 0,75 | No/ Pyridine nucleotide-disulphide oxidoreductase and L-lysine 6-monooxygenase (NADPH-requiring) | Cell redox homeostasis | | |
| PAAG_08580 | Disrupter of telomere silencing protein Dot5* | 0.59 | No/ No | Cell redox homeostasis | | |
| UNCLASSIFIED | | | | | | |
| PAAG_05168 | Predicted protein | 0.58 | No/ No | _ | | |
| PAAG_02524 | Predicted protein | 0.60 | No/ No | _ | | |
| PAAG_02145 | Predicted protein | 0.61 | No/ No | _ | | |
| PAAG_00604 | Putative uncharacterized protein | 0.61 | No/ GCN5-like domain | _ | | |
| PAAG_03358 | Predicted protein | 0.61 | No/ No | _ | | |
| PAAG_04601 | Conserved hypothetical protein | 0.63 | No/ No | _ | | |
| PAAG_12435 | Predicted protein | 0.63 | No/ No | _ | | |
| PAAG_02146 | Putative uncharacterized protein | 0.66 | No/ DUF3328 domain | _ | | |
| PAAG_03461 | Putative uncharacterized protein | 0.68 | No/ No | _ | | |
| PAAG_06898 | Putative uncharacterized protein | 0.73 | No/ No | _ | | |
| PAAG_03435 | Predicted protein | 0.74 | No/ No | _ | | |
| PAAG_06420 | Putative uncharacterized protein | 0.77 | No/ No | _ | | |
| PAAG_02832 | Predicted protein | 0.79 | No/ No | _ | | |
| PAAG_05423 | Predicted protein | 0.81 | No/ No | _ | | |
| PAAG_00683 | Putative uncharacterized protein | 0.82 | No/ No | _ | | |
| PAAG_01235 | Predicted protein | 0.99 | No/ No | _ | | |
| PAAG_06415 | Predicted protein | 1.22 | No/ No | _ | | |
| PAAG_02983 | Predicted protein | 1.26 | No/ No | _ | | |

^a^Accession number from *Paracoccidioides* genome database (<http://www.broadinstitute.org/annotation/genome/paracoccidioides_brasiliensis/MultiHome.html>).

^b^Annotation from *Paracoccidioides* genome database or by homology from NCBI database (<http://www.ncbi.nlm.nih.gov/>; *);

^c^Expression profiles in log2_fold change obtained from fold change selection method for differentially expressed transcripts using a Fisher exact test with a p-value of 0.001.

^d^Metal binding shows Cu, Fe or Zn proteins of the *Paracoccidioides* spp. isolate *Pb*01 (Tristão et al., 2015) found in our RNAseq. The specific domain predictions were found using the *Paracoccidioides* genome database (<http://www.broadinstitute.org/annotation/genome/paracoccidioides_brasiliensis/MultiHome.html>).

^e^Biological process according to MIPS (<http://pedant.helmholtz-muenchen.de/pedant3htmlview/pedant3view?Method=analysis&Db=p3_r48325_Par_brasi_Pb01>) and Uniprot database (http://www.uniprot.org/).
