# Supplementary material for: Paracoccidioides spp. ferrous and ferric iron assimilation pathways
Source: Front Microbiol. 2015 Aug 12;6:821. doi: 10.3389/fmicb.2015.00821 (PMC4585334; doi:10.3389/fmicb.2015.00821)
Supplement: Supplementary file 2 [file Table2.DOCX]

**Supp. Table 2. Some down-regulated transcripts of *Pb*01 yeast cells under iron deprivation for 24 h detected by RNAseq analysis.**

| ID^a^ | Annotation^b^ | Fold change (log2)^c^ | Metal binding/ Specifc domain^d^ | Functional Categories^e^ | | |
| --- | --- | --- | --- | --- | --- | --- |
| METABOLISM | | | | | | |
| Amino acid metabolism | | | | | | |
| PAAG_01419 | Kynureninase | -0.77 | No/ Aminotransferase class-V | Tryptophan degradation | | |
| PAAG_00397 | 5-aminolevulinate synthase | -0.75 | No/ Aminotransferase class I and II/ class-V; Cys/Met metabolism PLP-dependent enzyme | Heme biosinthethic process | | |
| PAAG_05776 | Dihydroxy-acid dehydratase | -0.72 | No/ Dehydratase family; ilvD: dihydroxy-acid dehydratase | Valin, leucine and Isoleucine biosynthesis | | |
| PAAG_05995 | [choline dehydrogenase*](http://www.ncbi.nlm.nih.gov/blast/Blast.cgi#alnHdr_225555325) | -0.69 | No/ GMC oxidoredutase domain; FAD binding domain | Glycine biosynthesis/ stress response | | |
| PAAG_06600 | Tyrosinase central domain-containing protein | -0.67 | No/ Common central domain of tyrosinase | Tyrosine degradation | | |
| Nitrogen and sulfur metabolism | | | | | | |
| PAAG_01321 | Oxidoreductase 2-nitropropane dioxygenase family | -0.63 | No/ IMP dehydrogenase_GMP reductase domain; Dihydroorotate dehydrogenase; Nitronate monooxygenase; FMN-dependent dehydrogenase. | | Nitrogen, sulfur and selenium metabolism | |
| C-compound and carbohydrate metabolism | | | | | | |
| PAAG_06863 | Mannitol dehydrogenase | -0.78 | No/ Zinc-binding dehydrogenase and Alcohol dehydrogenase GroES-like domain | | C-compound and carbohydrate metabolism | |
| Lipid, fatty acid and isoprenoid metabolism | | | | | | |
| PAAG_07746 | 3-ketoacyl-CoA thiolase | -0.93 | No/ N- and C-terminal thiolase domain; AcCoA-C-Actrans: acetyl-CoA C-acetyltransferase | | Lipid, fatty acid and isoprenoid metabolism | |
| PAAG_03407 | Delta(3,5)-delta(2,4)-dienoyl-CoA isomerase | -0.83 | No/ Enoyl-CoA hydratase and isomerase family | | Lipid, fatty acid and isoprenoid metabolism | |
| PAAG_01222 | Acyl-CoA dehydrogenase family protein | -0.82 | No/ Acyl-CoA dehydrogenase, N-, middle and C-terminal domain; Cytochrome b5-like Heme/Steroid binding domain | | Lipid, fatty acid and isoprenoid metabolism | |
| PAAG_03873 | Estradiol 17-beta-dehydrogenase | -0.82 | No/ Enoyl-(Acyl carrier protein) reductase; short chain dehydrogenase; KR domain | | Fatty acid metabolism | |
| PAAG_02042 | Phospholipase D | -0.59 | No/ No | | Phospholipid metabolic process | |
| Phosphate metabolism | | | | | | |
| PAAG_00017 | [catalytic protein kinase domain-containing protein*](http://www.ncbi.nlm.nih.gov/blast/Blast.cgi#alnHdr_295673865) | -0.96 | No/ Protein tyrosine kinase; kinase domain | Phosphate metabolism | | |
| PAAG_05991 | [beta-ketoacyl reductase*](http://www.ncbi.nlm.nih.gov/blast/Blast.cgi#alnHdr_225555321) | -0.69 | No/ Enoyl-(Acyl carrier protein) reductase; short chain dehydrogenase/ KR domain | Long-chain fatty acid biosynthethic process | | |
| ENERGY | | | | | |  |
| Electron transport and membrane-associated energy conservation | | | | | |  |
| PAAG_06103 | Succinate dehydrogenase iron-sulfur subunit | -0.71 | Yes (Fe)/ 2Fe-2S iron-sulfur cluster binding domain; 4Fe-4S dicluster domain; dhsB: succinate dehydrogenase and fumarate reductase iron-sulfur | Cellular respiration/ Mitochondrial electron transport, succinate to ubiquinone | | |
| PROTEIN FATE | | | | | |  |
| PAAG_03944 | Cytosolic Fe-S cluster assembling factor NBP35 | -0.68 | Yes (Zn)/ CobQ,CobB,MinD,ParA nucleotide binding domain; AAA domain; ParA/MinD ATPase like | Iron-sulfur cluster assembly | | |
| PAAG_12057 | [ubiquitin-conjugating enzyme E2*](http://www.ncbi.nlm.nih.gov/blast/Blast.cgi#alnHdr_240274693) | -0,60 | No/ No | Protein degradation | | |
| STRESS RESPONSE | | | | | |  |
| PAAG_03502 | Cytochrome c peroxidase | -0.72 | No/ Peroxidase domain | Oxidative stress response | | |
| PAAG_01454 | Catalase | -0.68 | No/ Catalase domain; Catalase-related immune-responsive | Oxidative stress response | | |
| PAAG_02926 | Superoxide dismutase | -0.58 | Yes (Fe)/ Iron, manganese superoxide dismutases C-terminal domain; Iron/manganese superoxide dismutases, alpha-hairpin | Detoxification | | |
| TRANSPORT | | | | | |  |
| PAAG_11860 | Nonspecific lipid-transfer protein* | -0.90 | No/ N- and C-terminal thiolase; 3-Oxoacyl-[acyl-carrier-protein (ACP)] synthase III | Transport routes | | |
| PAAG_07762 | Calcium transporter | -0.78 | Yes (Fe)/ VIT family | Homeostasis of metal ions (Na, K, Ca etc.) | | |
| PAAG_04554 | Fatty acid transporter protein | -0.75 | No/ AMP-binding enzyme C-terminal domain | Lipid transport | | |
| PAAG_01242 | Trichothecene efflux pump | -0.66 | No/ Major facilitator superfamily; Fungal trichothecene efflux pump (TRI12); Sugar (and other) | Drug/ toxin transport | | |
| MISCELLANEOUS | | | | | |  |
| PAAG_01243 | Phenylacetone monooxygenase | -1.04 | No/ Flavin-binding monooxygenase-like; Pyridine nucleotide-disulphide oxidoreductase | Oxidoreductase/aerobic aromate catabolism | | |
| UNCLASSIFIED | | | | | | |
| PAAG_03531 | Predicted protein | -1.72 | No/ No | _ | | |
| PAAG_01608 | Hypothetical protein | -1.48 | Yes (Zn)/ Zinc knuckle | _ | | |
| PAAG_07906 | Hypothetical protein | -1.39 | No/ No | _ | | |
| PAAG_03501 | Conserved hypothetical protein | -1.34 | No/ No | _ | | |
| PAAG_01100 | Predicted protein | -1.16 | No/ No | _ | | |
| PAAG_01244 | Beta-lactamase family protein* | -1.05 | No/ Beta-lactamase domain | _ | | |
| PAAG_02020 | Hypothetical protein | -0.88 | No/ No | _ | | |
| PAAG_05201 | Hypothetical protein | -0.86 | No/ No | _ | | |
| PAAG_09102 | Conserved hypothetical protein | -0.84 | No/ No | _ | | |
| PAAG_02925 | Hypothetical protein | -0.79 | No/ No | _ | | |
| PAAG_11066 | Hypothetical protein | -0.73 | No/ No | _ | | |
| PAAG_04375 | Hypothetical protein | -0.70 | No/ No | _ | | |
| PAAG_08188 | Predicted protein | -0.67 | No/ No | _ | | |
| PAAG_02996 | Hypothetical protein | -0.67 | No/ No | _ | | |
| PAAG_08554 | Predicted protein | -0.66 | No/ No | _ | | |
| PAAG_06033 | Conserved hypothetical protein | -0,65 | No/ No | _ | | |
| PAAG_05517 | Predicted protein | -0.60 | No/ No | _ | | |
| PAAG_02349 | Predicted protein | -0.59 | No/ No | _ | | |
| PAAG_07345 | Putative uncharacterized protein | -0.57 | No/ No | _ | | |

^a^Accession number from *Paracoccidioides* genome database (<http://www.broadinstitute.org/annotation/genome/paracoccidioides_brasiliensis/MultiHome.html>).

^b^Annotation from *Paracoccidioides* genome database or by homology from NCBI database (<http://www.ncbi.nlm.nih.gov/>; *);

^c^Expression profiles in log2_fold change obtained from fold change selection method for differentially expressed transcripts using a Fisher exact test with a p-value of 0.001.

^d^Metal binding shows the Cu, Fe or Zn proteins of the *Paracoccidioides* spp. isolate *Pb*01 (Tristão et al., 2015) found in our RNAseq. The specific domain predictions were found using the *Paracoccidioides* genome database (<http://www.broadinstitute.org/annotation/genome/paracoccidioides_brasiliensis/MultiHome.html>).

^e^Biological process according to MIPS (<http://pedant.helmholtz-muenchen.de/pedant3htmlview/pedant3view?Method=analysis&Db=p3_r48325_Par_brasi_Pb01>) and Uniprot database (<http://www.uniprot.org/>).
