# Supplementary figures and images for: Paracoccidioides spp. ferrous and ferric iron assimilation pathways
Source: Front Microbiol. 2015 Aug 12;6:821. doi: 10.3389/fmicb.2015.00821 (PMC4585334; doi:10.3389/fmicb.2015.00821)

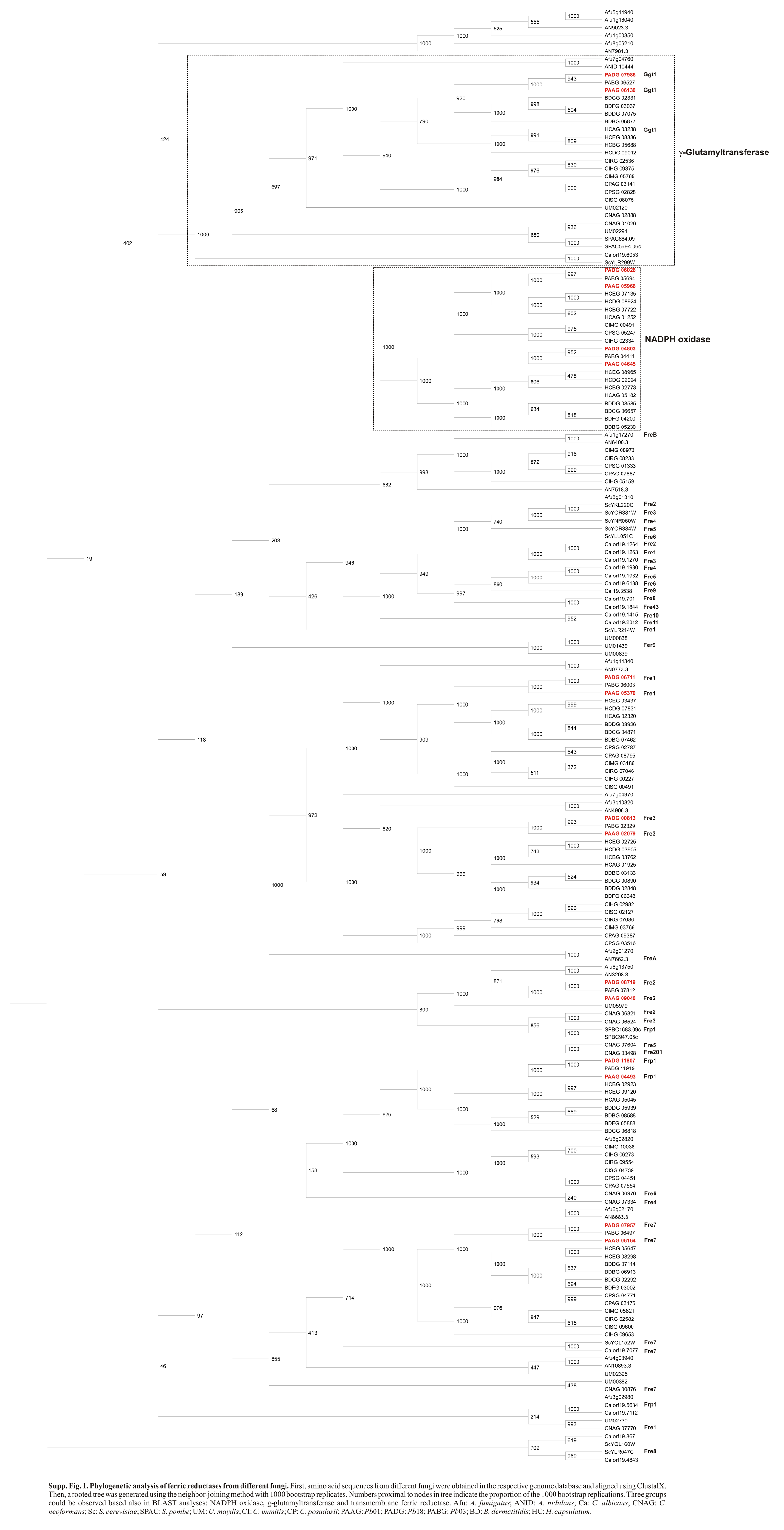

Supplement: Supplementary file 3 [file Image1.TIF]

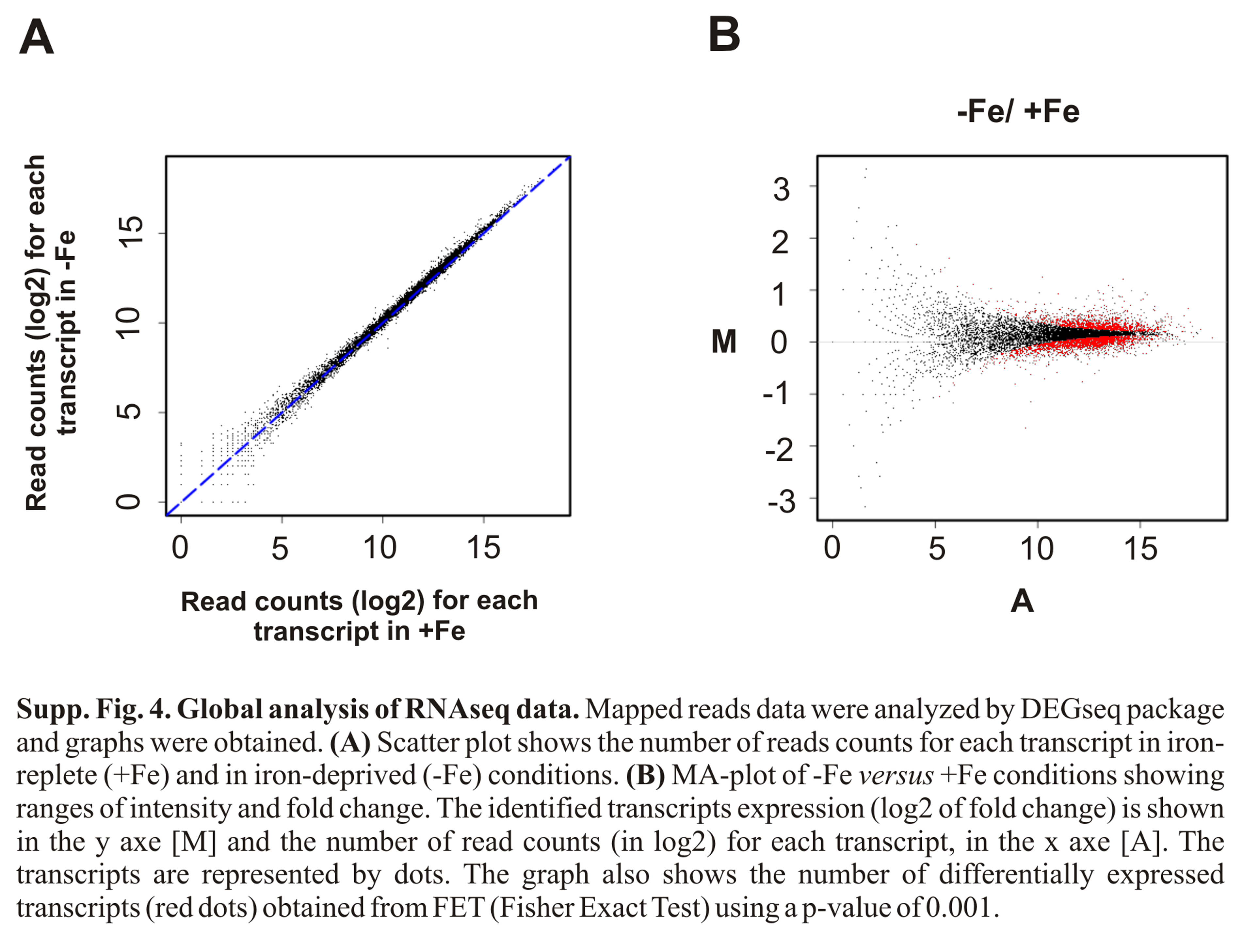

Supplement: Supplementary file 6 [file Image4.TIF]
