# Supplementary material for: Paracoccidioides spp. ferrous and ferric iron assimilation pathways
Source: Front Microbiol. 2015 Aug 12;6:821. doi: 10.3389/fmicb.2015.00821 (PMC4585334; doi:10.3389/fmicb.2015.00821)
Supplement: Supplementary file 4 [file Image2.PDF]

```
Afu7g04760          ------------------------------------------------------------
ANID_10444          ------------------------------------------MQLQGYDGNAQDVEKQPL 18
UM02291             ---MTALRSAHRP-SSLRSTANSEAGSIEP--------------IQEEVVSNDGQISAAA 42
SPAC664.09          ---------------------------------------------------MGINTSSAQ 9
CNAG_01026          MPSIAASSPDHDENAPLLASPTLEAGTTRPPTPSPPWQFWNRPPFRRVHFAEHDQFVPPE 60
SPAC56E4.06c        -------------------------------------------------------MSPTD 5
Ca_orf19.6053       -----------------------------------------------------MKSEIPP 7
ScYLR299W           --------------------------------------------------MLLCNRKVPK 10
BDCG_02331          ------------------------------------------------------------
BDFG_03037          ------------------------------------------------------------
PAAG_06130          ------------------------------------------------------------
HCDG_09012          ------------------------------------------------------------
UM2120              ----------------MSLQGRRDLKAAYLTQRDNPCPPNHKSRIRSNSVWSRAQRQSIT 44
HCBG_05688          ------------------------------MLRLTFLMDQLIDTIQNMALDAIADMTFTQ 30
CNAG_02888          ------------------------------------------------------------
BDDG_07075          ------------------------------------------------------------
BDBG_06877          ------------------------------------------------------------
HCAG_03238          ------------------------------------------------------------
HCEG_08336          ------------------------------------------------------------
CPSG_02828          ------------------------------------------------------------
CIMG_05765          ------------------------------------------------------------
CIRG_02536          ------------------------------------------------------------
CISG_06075          ------------------------------------------------------------
PADG_07986          ------------------------------------------------------------
PABG_06527          ------------------------------------------------------------
CIHG_09375          ------------------------------------------------------------
CPAG_03141          ------------------------------------------------------------


Afu7g04760          ------------------------------------------------------------
ANID_10444          LPSLDNELQVSFSLQNQTRNSGSTMSPSPSLSLSRLLRLSVCTTLCLVLVVFHVPSVLPS 78
UM02291             TPDTETSALLPSHRHRTQLLAPPSSHKSRSRTIWNSILVLLAIFTFAVTISIVLKNLLGE 102
SPAC664.09          SSGAASIARSSVNVKSGNRHLSSNKKSATSALEERASRPSILVTFLVLAGTILSLYIWPI 69
CNAG_01026          SVHSESEISADGNERYGTNPKYPRQRLQQEEKMGRWMMYCLLVLVGMVFGTLISRNWQNK 120
SPAC56E4.06c        TTPLLYSWDDQSRHQDPDWHKLRN---YHGAWYRRISRRRFSQFIFAFGLMTLFVLVYSI 62
Ca_orf19.6053       IKNTNYNCKIPRSTFFVVCVFSLL-----IYSLSTIYRSSGLVLPPLS-FIDQIPENLSR 61
ScYLR299W           TLNTCFILHIFTLLTLGVLVSGMPSKMVSFASQETLQRINNLLRGSANRDVDIIAEYLKK 70
BDCG_02331          ------------------------MLTGFLALLTSIAITVLLTSFYLFSSFTTFTETWGA 36
BDFG_03037          ------------------------MLTGFLALLTSIAITVLLTSFYLFSSFTTFTETWGA 36
PAAG_06130          ------------------------MLTGLLATVS-FAAMMLLTAFYLFSSIATFRVAWSV 35
HCDG_09012          ------------------------------------------------------------
UM2120              NADFEPTWRDDRLVTQSTLASPTHVVSISLVLLTLTYKNCAWRSSFLASSCNQRAATVES 104
HCBG_05688          GPIVNSSFCYGYVDVAYTLDMICRMFSNSLVVVVSATSMMLLIAVSLYSSLTTFWGRLDV 90
CNAG_02888          ------------------------------------------------------------
BDDG_07075          ------------------------MLTGFLALLTSIAITVLLTSFYLFSSFTTFTETWGA 36
BDBG_06877          ------------------------MLTGFLALLTSIAITVLLTSFYLFSSFTTFTETWGA 36
HCAG_03238          ------------------------MFNHSLVVVVSAASMMLLIAVSLHSSLTTFWGRLDV 36
HCEG_08336          ------------------------MFSHSLVVVVSAASMMLLIAVSLYSSLTTFWGRLDV 36
CPSG_02828          --------------------------------MISPAVLALCVGFYIFSTFADLSCATPI 28
CIMG_05765          -------------------------MLRSLLIMISPAVLALCVGFYIFSTFADLSCATPI 35
CIRG_02536          -------------------------MLRSLLIMISPAVLALCVGFYIFSTFADLSCATPI 35
CISG_06075          ------------------------------------------------------------
PADG_07986          ------------------------MLTGLLATVS-FAAMMLLTAFYRFSSIATFRAAWNV 35
PABG_06527          ------------------------MLTGLLAPVS-FAAMMLLTAFYLFSSIATFRVAWNV 35
CIHG_09375          -------------------------MLRSLLIMISPAVLALCVGFYIFSTFADLSCATPI 35
CPAG_03141          -------------------------MLRSLLIMISPAVLALCVGFYIFSTFADLSCATPI 35
```

Afu7g04760	--	
ANID_10444	P-------LDSYDRYTRAHKYERSAEAHD-------------------------------	100
UM02291	FNDPEDPSLPIDPPGTDPRGR-RHPAVLAT------------------------------	131
SPAC664.09	LSPDLFFANQRCSFKYKNKGSQRVVV----------------------------------	95
CNAG_01026	GVTGDMPMVPPVWTLPPPTGLPRNDAYLIN------------------------------	150
SPAC56E4.06c	SS-NLHTPTQFTGHKVR---	78
Ca_orf19.6053	SDPYLTLHVDISDDPLSDKIGQPELNPSPK------------------------------	91
ScYLR299W	DDDDDGGDKDHHNIDIDPLPRRPSLTPDRQ------------------------------	100
BDCG_02331	K-------LSGSGGDPGKYGNCGGHHHG--------------------------------	57
BDFG_03037	K-------LSGSGGDPGKYGNCGGHHHG--------------------------------	57
**PAAG_06130**	S-------SGQGQAKTGFDGTPGNEDVPG-------------------------------	57
HCDG_09012	--	
UM2120	SGMQLRNDLSTWAAILGALVLTAGSQTSASPIHGIVDRESRNNGKQPSFYGAASQGNSAE	164
HCBG_05688	Q-------LSNDASAPR-NGRHG-------------------------------------	105
CNAG_02888	--	
BDDG_07075	K-------LSGSGGDPGKYGNCGGHHHG--------------------------------	57
BDBG_06877	K-------LSGSGGNPGKYGNCGGHHHG--------------------------------	57
HCAG_03238	Q-------LSSDASPPG-NGRHG-------------------------------------	51
HCEG_08336	Q-------LSSDASAPR-NGRHG-------------------------------------	51
CPSG_02828	RRHSSYPWLNQGSGRPDSGQEVDAWARQD-------------------------------	57
CIMG_05765	RRHSSYPWLNQGSGRPDSGQEVDAWARQD-------------------------------	64
CIRG_02536	RRHSSYPWLNQGSGRPDSGQEVDAWARQD-------------------------------	64
CISG_06075	--	
**PADG_07986**	S-------SGQGQAKTGPDGTPGNEDAPG-------------------------------	57
**PABG_06527**	S-------SGQGQAKTGSDGTPGNEDAPG-------------------------------	57
CIHG_09375	RRHSSYPWLNQGSGRPDSGQEVDAWARQD-------------------------------	64
CPAG_03141	RRHSSYPWLNQGSGRPDSGQEVDAWARQD-------------------------------	64

Afu7g04760	--	
ANID_10444	----GKRGAVASESAICSRHG**TDIILMG**---**GNAADA**---------**MVATMLCVGVVG**--	142
UM02291	----GRKAGVATENEICSRIG**MDILLAK**---**GTAVDA**---------**AVASTFCVGVLN**--	173
SPAC664.09	---EGKNGVVATEEETCSQIG**VGILKAG**---**GNAVDA**---------**AIASGICIGAVN**--	138
CNAG_01026	----ATTAAVASEDVTCSNLG**LSILQDKN**--**GSAVDA**---------**AITTTLCIGLLN**--	193
SPAC56E4.06c	----GRRGAVASEVPVCSDIG**VSMLADG**---**GNAVDA**---------**AIASTFCIGVVN**--	120
Ca_orf19.6053	HLHVGSKAMVASDVPLCSTMGK**-EILLR**--**GGNAADA**---------**AVTVALCIGSVN**--	137
ScYLR299W	LLKVGLHGAISSDLEVCSNLTI**NEVLLKFPGSNAADA**---------**AVTQALCKGMVN**--	149
BDCG_02331	-----ELGAVASENRNCSRLG**GEMLKIG**---**GNAADA**---------**MVA*TVFCIGVT***---	97
BDFG_03037	-----ELGAVASENRNCSRLG**GEMLKIG**---**GNAADA**---------**MVA*TVFCIGVT***---	97
**PAAG_06130**	----RSLGAVASENGYCSGLG**VGMLKEG**---**GNAVDA**---------**MVATVFCIGVT**---	98
HCDG_09012	--**MVATVFCIGVI**---	11
UM2120	YSVEGKHGAVSSEVDVCSNIG**AQLLQQG**---**GSAVDS**---------**IIGTALCVGSIA**--	210
HCBG_05688	-----QLGAVASENRNCSQMG**AEMLKMG**---**GTAADA**---------**MVATVFCIGVV**---	145
CNAG_02888	----MPSGAVTSEQIRASDIG**TTILKAG**---**GSAADA**---------**IIATTLAVNTLS**--	42
BDDG_07075	-----ELGAVASENRNCSRLG**GEMLKIG**---**GNAADA**---------**MVA*TVFCIGVT***---	97
BDBG_06877	-----ELGAVASENRNCSRLG**GEMLKIG**---**GNAADA**---------**MVATVFCIGVT**---	97
HCAG_03238	-----QLGAVASENRNCSQMG**AEMMKMG**---**GTAADADMNSFDLPQMVATVFCIGVI**---	100
HCEG_08336	-----QLGAVASENRNCSQMG**AEMLKMG**---**GTAADADMNSFDLPQMVATVFCIGVI**---	100
CPSG_02828	----DRLGAVASESSICSGFG**VDMLKLG**---**GNAADA**---------**MVATVFCVGVVG**--	99
CIMG_05765	----DRLGAVASESSICSGFG**IDMLK*LG***---***GNAADA***---------***MVATVFCVGVVG***--	106
CIRG_02536	----DRLGAVASESSICSGFG**IDMLK*LG***---***GNAADA***---------***MVATVFCVGVVG***--	106
CISG_06075	--	
**PADG_07986**	----RGLGAVASENGYCSGLG**VGMLKEG**---**GNAVDA**---------**MVATVFCIGVTGEP**	101
**PABG_06527**	----RGLGAVASENGYCSGLG**VGMLKEG**---**GNAVDA**---------**MVATVFCIGVTGEP**	101
CIHG_09375	----DRLGAVASESSICSGFG**IDMLK*LG***---***GNAADA***---------***MVATVFCVGVVG***--	106
CPAG_03141	----DRLGAVASESSICSGFG**VDMLKLG**---**GNAADA**---------**MVATVFCVGVVG**--	106

```
Afu7g04760          -----------------------MYHSGIGGGGFMLVRAPNG------------------ 19
ANID_10444          -----------------------MYHSGIGGGGFMLVKSPDG------------------ 161
UM02291             -----------------------MFSSGIGGGGFMIVRDPSACSAKGAKQPDC------- 203
SPAC664.09          -----------------------SFSSGIGGGGFMLIRHPNG------------------ 157
CNAG_01026          -----------------------AFSSGIGGGGFMVVRVPETHEIKDQVLRDIGYDDELG 230
SPAC56E4.06c        -----------------------FFSSGIGGGGFMLIKHPNE------------------ 139
Ca_orf19.6053       -----------------------SHSSGIGGGGFIVS--RNNGD---------------- 156
ScYLR299W           -----------------------FFNSGIGGGGYVVFSGKDDED---------------- 170
BDCG_02331          ----------------------AMYHSGIGGGGFAVVRTPDD------------------ 117
BDFG_03037          ----------------------AMYHSGIGGGGFAVVRTPDD------------------ 117
PAAG_06130          ----------------------AMYHSNIGGGGFAVVRTPEN------------------ 118
HCDG_09012          ----------------------AMYHSGISGGGFAIVRTPEG------------------ 31
UM2120              -----------------------SYHSGLGGGGHAVLRSPITNDDRTNRKRSNPNDARSK 247
HCBG_05688          ----------------------AMYHSGISGGGFAIVRTPEG------------------ 165
CNAG_02888          -----------------------PYHSDIGGGGFAIVKEPGD------------------ 61
BDDG_07075          ----------------------AMYHSGIGGGGFAVVRTPDD------------------ 117
BDBG_06877          ----------------------AMYHSGIGGGGFAVVRTPDD------------------ 117
HCAG_03238          ----------------------AMYHSGISGGGFAIVRTPGG------------------ 120
HCEG_08336          ----------------------AMYHSGISGGGFAIVRTPEG------------------ 120
CPSG_02828          -----------------------MYHSGIGGGGFMLIRSPDD------------------ 118
CIMG_05765          -----------------------MYHSGIGGGGFMLIRSPDD------------------ 125
CIRG_02536          -----------------------MYHSGIGGGGFMLIRSPDD------------------ 125
CISG_06075          ------------------------------------------------------------
PADG_07986          PSTSYIILGQICSLADRIIALLAMYHSNIGGGGFAVVRTPEN------------------ 143
PABG_06527          PSTSYIILGQICSLADRIIALLAMYHSNIGGGGFAVVRTPEN------------------ 143
CIHG_09375          -----------------------MYHSGIGGGGFMLIRSPDD------------------ 125
CPAG_03141          -----------------------MYHSGIGGGGFMLIRSPDD------------------ 125
```

```
Afu7g04760          --SFEFIDFRETAPAAAFEEMFNNS---THASTIGGLARRTNVSLSGVPGELRGLEYLHK 74
ANID_10444          --SFEYIDFRETAPAAAYETMFNNL---TDASTLGGLA-------SGVPGELRGLELLHS 209
UM02291             -IEHTTIDFRETAPAAANKTMYVGRVP---KAQFGGLA-------VGVPGELRGLQEAHK 252
SPAC664.09          --TAHSLNFRETAPAGASKNMFHGN---STLSQVGGLS-------VAVPGEIAGYERAWK 205
CNAG_01026          ESRVVALDFRETSPALCEKDTYGTHKAGRMAAQVGGLA-------IGVPGELRGLEAAHK 283
SPAC56E4.06c        --TAQSLTFREIAPGNVSKHMFDKN---PMLAQVGPLS-------IAIPGELAGLYEAWK 187
Ca_orf19.6053       ---AISIDAREMAPGSAYKEMYGNS---LVLSKIGGLS-------IAIPGELKGLYELFK 203
ScYLR299W           ---HLSIDFREKAPMDSHKFMFENC---SLCSKIGGLA-------VGVPGELMGLYRLFK 217
BDCG_02331          --KYEFIDFREVAPAAAFEDMYKDN---VEGSIIGGLA-------SGVPGEVRGLEYLHK 165
BDFG_03037          --KYEFIDFREVAPAAAFEDMYKDN---VEGSIIGGLA-------SGVPGEVRGLEYLHK 165
PAAG_06130          --RYEFVDFRETAPAAAFEDMYKDN---EQASITGGLA-------SGVPGEVRGLEYLHK 166
HCDG_09012          --EYEVVDFREMAPAAAHKDMYKGN---VEGAVLGGLA-------SAVPGEIRGLQYLHE 79
UM2120              TSTYVHIDFREVFPAAATEDMYTNNPV-KNASLYGGLA-------VGVPGEPKAWWDLHQ 299
HCBG_05688          --GYEVVDFREMAPATAHKDMYKGN---VEGAVLGGLA-------SAVPGEIRGLQYLHE 213
CNAG_02888          SGQVKCLDFRQCAPEGATPELFKTS---DTSTSVGGLA-------VAVPGELKGLEELHK 111
BDDG_07075          --KYEFIDFREVAPAAAFEDMYKDN---VEGSIIGGLA-------SGVPGEVRGLEYLHK 165
BDBG_06877          --KYEFIDFREVAPAAAFEDMYKDN---VEGSIIGGLA-------SGVPGEVRGLEYLHK 165
HCAG_03238          --EYEVVDFREMAPAAAHKDMYKGN---VEGAVLGGLA-------SAVPGEIRGLQYLHE 168
HCEG_08336          --EYEVVDFREMAPAAAHKDMYKGN---VEGAVLGGLA-------SAVPGEIRGLQYLHE 168
CPSG_02828          --TYEFVDFRETAPAAAFQDMYNNN---TDASIFGGLA-------SGVPGEVRGLEYLHK 166
CIMG_05765          --TYEFVDFRETAPAAAFQDMYNNN---TDASIFGGLA-------SGVPGEVRGLEYLHK 173
CIRG_02536          --TYEFVDFRETAPAAAFQDMYNNN---TDASIFGGLA-------SGVPGEVRGLEYLHK 173
CISG_06075          ------------------------------------------------------------
PADG_07986          --RYEFVDFRETAPAAAFEDMYKDN---EQASITGGLA-------SGVPGEVRGLEYLHK 191
PABG_06527          --RYEFVDFRETAPAAAFEDMYKDN---EQASITGGLA-------SGVPGEVRGLEYLHK 191
CIHG_09375          --TYEFVDFRETAPAAAFQDMYNNN---TDASIFGGLA-------SGVPGEVRGLEYLHK 173
CPAG_03141          --TYEFVDFRETAPAAAFQDMYNNN---TDASIFGGLA-------SGVPGEVRGLEYLHK 173
```

```
Afu7g04760          KYG--SLPWSVLVQPAIKTAREGFPVGQDLVKYMKSAVGDGI------DFLVENPTWALD 126
ANID_10444          KYG--SLPWSVLVQPAINTARNGFPVGQDLVRYMDSAVGDGE------DFLVNDPSFAVD 261
UM02291             RYG--RLAWKRLVQPSVELAKS-ATVSKELERRLSFFG----------GFIYDEPVWREI 299
SPAC664.09          MYG--SLPWHKLFEPTIRLMRDGMPMPKELASRIRRPE---------FSYFKTHPDWSKI 254
CNAG_01026          LYG--ALPWKDVVMPVAELAKG-WRVSRELARRLRLFG----------DFMLSSPTWSAV 330
SPAC56E4.06c        SHG--LLDWSKLLEPNVKLAREGFPVTRAMERVLKLPE---------MAHLLKDPIWQPI 236
Ca_orf19.6053       LHGSGNLSWKQLFEPVIKLNRNGFKCSKIFETVLAKEYDLVLSR---VPVLKDSWDFIFK 260
ScYLR299W           ERGSGQVDWRDLIEPVAKLGSVGWQIGEALGATLELYEDVFLT-------LKEDWSFVLN 270
BDCG_02331          KYG--VLPWSTVMQPAIRTARDGWPVNEDLVHYMEAETAGEN------DFLSKDPAWAVD 217
BDFG_03037          KYG--VLPWSTVMQPAIRTARDGWPVNEDLVHYMEAETAGEN------DFLSKDPAWAVD 217
PAAG_06130          KYG--SLPWPKVMQPAIRTARDGWRVNEDLARMMESETKDE-------DFLSKDPSWAID 217
HCDG_09012          KYG--TLPWSTVMQPAIHLAREGWPINVDLVNYMETATAGKE------DFLSKDPSWAVD 131
UM2120              KYG--KLEWSKVFEPAITLNRRGFKVTAELAKALNTT---QY------PFLCKDKRWSRF 348
HCBG_05688          KYG--TLPWSTVMQPAIRLAREGWPVNVDLVNYMETATAGKE------DFLSKDPSWAVD 265
CNAG_02888          EYG--VLPWSRLFKESIELAEEGMEVRGDLYDFITREANPAGSSNIRGTWMMEDPTYVSL 169
BDDG_07075          KYG--VLPWSTVMQPAIRTARDGWPVNEDLVHYMEAETAGEN------DFLSKDPAWAVD 217
BDBG_06877          KYG--ILPWSTVMQPAIRTARHGWPVNEDLVHYMEADTAGEN------DFLSKDPAWAVD 217
HCAG_03238          KYG--TLPWSTVMKPAIRLAREGWPVNVDLVNYMETATAGKE------DFLSKDPSWAVD 220
HCEG_08336          KYG--TLPWSTVMQPAIHLAREGWPINVDLVNYMETATAGKE------DFLSKDPSWAVD 220
CPSG_02828          KHG--LLPWRLVMQPAIHTARYGFPVTEDLVRYMREATEGKE------DFLTNNPTWALD 218
CIMG_05765          KHG--LLPWRLVMQPAIHTARYGFPVTEDLVRYMREATEGKE------DFLTNNPTWALD 225
CIRG_02536          KHG--LLPWRLVMQPAIHTARYGFPVTEDLVRYMREATEGKE------DFLTNNPTWALD 225
CISG_06075          ------------MQPAIHTARYGFPVTEDLVRYMREATEGKE------DFLTNNPTWALD 42
PADG_07986          KYG--SLPWPKVMQPAIRTARDGWRVNEDLARMMESETKDE-------DFLSKNPSWAID 242
PABG_06527          KYG--SLPWPKVMQPAIRTARDGWRVNEDLARMMESETKDE-------DFLSKNPSWAID 242
CIHG_09375          KHG--LLPWRLVMQPAIHTARYGFPVTEDLVRYMREATEGKE------DFLTNNPTWALD 225
CPAG_03141          KHG--LLPWRLVMQPAIHTARYGFPVTEDLVRYMREATEGKE------DFLTNNPTWALD 225
                                .                :   :                :     :


Afu7g04760          FAPN--GTRLGLGDTMTRRRYADTLETIANKGPAAFYSGPIAETMINALQ---AANGTMT 181
ANID_10444          FAPN--GERVKLGDTITRKRYAKTLETIAAEGPDAFYSGPIAEYTIKALQ---AANGTMT 316
UM02291             FVDDNTGQLKREGDTFHRPAYAQTLQSIADHGPDVFYSGAIAESLVRTTQ---AHGGILT 356
SPAC664.09          FAPE--GVFLHVGEKFYRPALASTLEEIAKFGPEVFYTGKIAERLVKFVQ---QQGGILT 309
CNAG_01026          YAPR--GPLLVEGDFIQRLNYGKTLEKIAEEGASAFYQGEIAESSVKTIA---KAGGVMT 385
SPAC56E4.06c        LMPN--GKVLRAGDKMFRPAYAKTLEIIANKGIEPFYRGELTNSMVKFIQ---DNGGIVT 291
Ca_orf19.6053       PNGE----LLQEGDVITRPNYANTLELIANNGSSSIFYDPNGPIVQSLVSTIQKWGGIAT 316
ScYLR299W           STHDG---VLKEGDWIKRPALSNMLMELAKNGSVAPFYDPDHWIAKSMIDTVAKYNGIMN 327
BDCG_02331          FAPN--GVLLGLGDIITRRRYADTLEKIAQYGADAFYSGPIAQATIRALQ---AQNGTMT 272
BDFG_03037          FAPN--GVLLGLGDIITRRRYADTLEKIAQYGADAFYSGPIAQATIRALQ---AQNGTMT 272
PAAG_06130          FAPN--GTRLGLGDIITRRRYARTLEQIANHGPDAFYSGPIAEATIQALQ---ASNGTMT 272
HCDG_09012          FAPN--GTRLGLGDTITRARYSQTLETIANYGPDAFYSGPLADSMIRALQ---AHNGTMT 186
UM2120              YCVN--GKVAQLGDTIKKERFAKTLELIASKGVDPYYYGEIADDIVDTIANNKVLKGILT 406
HCBG_05688          FAPN--GTRLGLGDTITRARYSQTLETIANYGPDAFYSGPLADSMIRALQ---AHNGTMT 320
CNAG_02888          ITKD--GQAIPIGSTWKRPEYAKTLRKIAEEGAAAFYQGEIAEGLVKAVR---ARDGVMT 224
BDDG_07075          FAPN--GVLLGLGDIITRRRYADTLEKIAQYGADAFYSGPIAQATIRALQ---AQNGTMT 272
BDBG_06877          FAPN--GVLLGLGDIITRRRYADTLEKIAKYGADAFYSGPIAQATIRALQ---AQNGTMT 272
HCAG_03238          FAPN--GTRLGLGDTITRTRYSQTLETIANYGPDAFYSGPLADSMIRALQ---AHNGTMT 275
HCEG_08336          FAPN--GTRLGLGDTITRARYSQTLETIANYGPDAFYSGPLADSMIRALQ---AHNGTMT 275
CPSG_02828          FAPN--GTRLGLGDTITRKRYANTLETIANYGADAFYSGAIAETIIQALH---AQNGTMT 273
CIMG_05765          FAPN--GTRLGLGDTITRKRYANTLETIANYGADAFYSSAIAETMIQALQ---AQNGTMT 280
CIRG_02536          FAPN--GTRLGLGDTITRKRYANTLETIANYGADAFYSGAIAETMIQALQ---AQNGTMT 280
CISG_06075          FAPN--GTRLGLGDTITRKRYANTLETIANYGADAFYSGAIAETMIQALQ---AQNGTMT 97
PADG_07986          FAPN--GTRLGLGDIITRRRYARTLEQIANHGPDAFYSGPIAEATIQALQ---ASNGTMT 297
PABG_06527          FAPN--GTRLGLGDIITRRRYARTLEQIANHGPDAFYSGPIAEATIQALQ---ASNGTMT 297
CIHG_09375          FAPN--GTRLGLGDTITRKRYANTLETIANYGADAFYSGAIAETMIQALQ---AQNGTMT 280
CPAG_03141          FAPN--GTRLGLGDTITRKRYANTLETIANYGADAFYSGAIAETIIQALH---AQNGTMT 280
                                *.   :   .  *  :*  *    : .                 *  .
```

```
Afu7g04760          MEDLRNYTVAIRNVSQIDYRG---------YQITSTSAPSSGTVALSILKILSTY----- 227
ANID_10444          LEDLRNYTAVVRDYSQINYRG---------YQVTSTTTPSSGSVAMNILKVLDTY----- 362
UM02291             LQDLHDYKVIVRPALQGSWLG---------KKVYTTHAPTSGPILLSILNMLSLIP---- 403
SPAC664.09          MEDMANFSVVVEEPIYGNFYD---------REVITCGSPCSGEALILGLNVLSKVDLSEG 360
CNAG_01026          LDDLKSFKAFSYPAIHSTFMS---------KDIYTTSAPSSGGIMLGLLNVLEPLN---- 432
SPAC56E4.06c        VEDFGNYSTVFADALHTSYRG---------HDVYTCTLPTSGPALIEGLNILDGYPLNTP 342
Ca_orf19.6053       VQDFSNYEVNLEKPLISTINN---------YTFYTSNGISSGLGLLAGLNFFDRV----- 362
ScYLR299W           LQDVSSYDVHVTKPLSMKIRKGANFIPDNDMTVLTSSGSSSGAALLAALRIMDNF----- 382
BDCG_02331          LDDLKNYTVAIRGISQVSYRG---------YKVTSTSAPSSGVVTMSILNILNEY----- 318
BDFG_03037          LDDLKNYTVAIRGISQVSYRG---------YKVTSTSAPSSGVVTMSILNILNEY----- 318
PAAG_06130          LGDLKNYTVTIRNVSQIMYRG---------YRVTGASAPSGGSVGLSILNILNQY----- 318
HCDG_09012          LDDLKNYSVVIRNASHINYRG---------YTVTSTRAPSSGVVALSILNIVGQY----- 232
UM2120              KHDLANYKVEYRNPRNVTLRDGK-------YRLFGTVAPSSGSVVLSTLQTVDQFRV--- 456
HCBG_05688          LDDLKNYSVVIRNASHINYRG---------YTVTSTRAPSSGVVALSILNIVGQY----- 366
CNAG_02888          VDDLENYKLKWREPLSTKFKD---------YTLYAPPAPASGAIWLSVMGMLSQF----- 270
BDDG_07075          LDDLKNYTVAIRGISQVSYRG---------YKVTSTSAPSSGVVTMSILNILNEY----- 318
BDBG_06877          LDDLKNYTVAIRGISQVSYRG---------YKVTSTSAPSSGVVTMSILNILNEY----- 318
HCAG_03238          LDDLKNYSVVIRNASHINYRG---------YTVTSTRAPSSGVVALSILNIVGQY----- 321
HCEG_08336          LDDLKNYSVVIRNASHINYRG---------YTVTSTRAPSSGVVALSILNIVGQY----- 321
CPSG_02828          LEDLKNYTVDIKETAQIDYRG---------FKVTSTSAPSSGAILLSTLNILEGY----- 319
CIMG_05765          LEDLKNYTVDIKETAQIDYRG---------FKVTSTSAPSSGAILLSTLNILEGY----- 326
CIRG_02536          LEDLKNYTVDIKETAQIDYRG---------FKVTSTSAPSSGAILLSTLNILEGY----- 326
CISG_06075          LEDLKNYTVDIKETAQIDYRG---------FKVTSTSAPSSGAILLSTLNILEGY----- 143
PADG_07986          LGDLKNYTVMIRNVSQIMYRG---------YRVTGASAPSGGSVGLSILNILNQY----- 343
PABG_06527          LGDLKNYTVMIRNVSQIMYRG---------YRVTGASAPSGGSVGLSILNILNQY----- 343
CIHG_09375          LEDLKNYTVDIKETAQIDYRG---------FKVTSTSAPSSGAILLSTLNILEGY----- 326
CPAG_03141          LEDLKNYTVDIKETAQIDYRG---------FKVTSTSAPSSGAILLSTLNILEGY----- 326
                      *. .:                         .       .*   :  :  .


Afu7g04760          DGFFAPGNVNLSTHRLDEAMRFGYET-KRTNLGDPLFVAGLD-EFEENMLKQSTIDEIRR 285
ANID_10444          EPLFTPENVNLSTHRLDEAMRFAYG--LRTVLGDPGFVDGMS-EYERDMISQQTADEIHS 419
UM02291             DFTSIGQVTSLNMHRFIEALKFGFG--QRTELADPAFMSSAGLERMSQIPTMSEALAIVP 461
SPAC664.09          TSILGCEMTDIGVHHLIETMKWMSA--GRTVLADPTFY--NNTDHVEQLLSLEYADEIRN 416
CNAG_01026          ITSNNGLKNPLNLHRFIEALKFAFG--ARSWVTDPAFAKDK--KRLEEVYTKEWADEIRK 488
SPAC56E4.06c        SLAF-----PKRLHLEVEAMKWLSA--GRTQFGDPDFLPLDHLDVVSKLLSKEFASQIRN 395
Ca_orf19.6053       FNE--SDDDTLFTHKLVESFKWLSSIRTRFGDID--NRQDLIDKYTNSTWIDDVLDEKKY 418
ScYLR299W           QNQEGGDYEKETTYHLLESMKWMASARSRLGDFEGEALPKHIEEVLDPEWALKAVKSIKR 442
BDCG_02331          DDFFAPGNVNLSTHRLAEAMRFAYG--QRTQLGDPSFVKRLA-EFEDHMLNASTAAMIRA 375
BDFG_03037          DDFFAPGNVNLSTHRLAEAMRFAYG--QRTQLGDPSFVKRLA-EFEDHMLNASTAAMIRA 375
PAAG_06130          DRFFSPGTVNLSTHRMTEAYRFAYG--QRTYLGDPSFVDNVT-EYEEDMLRVSTAAQIRA 375
HCDG_09012          DDFFVKGNVNLSTHHLVEAMRFAYG--Q---------------------PRNVCANPKP 268
UM2120              DGEEDLSNVNVSTHRLIEANKFSYG--QRTNYGDPGFVKNVS-RLEGEYLELPFSKENKN 513
HCBG_05688          DDFFVKGNVNLSTHHLVEAMRFAYG--Q-----------GMD-EYEDHMLNSSMAAIIRN 412
CNAG_02888          -EPAGYGSVT-DLHRLTEALRLAYG--QRTALGDPAYVDGVE-EKQRDWLTEERIKARAG 325
BDDG_07075          DDFFAPGNVNLSTHRLAEAMRFAYG--QRTQLGDPSFVKRLA-EFEDHMLNASTAAMIRA 375
BDBG_06877          DDFFAPGNVNLSTHRLAEAMRFAYG--QRTQLGDPSFVKRLA-EFEDHMLNASTAAMIRA 375
HCAG_03238          DDFFVKGNVNLSTHHLVEAMRFAYG--QRTHLADPSYSEGMD-EYEDHMLNSSMAAIIRN 378
HCEG_08336          DDFFVKGNVNLSTHHLVEAMRFAYG--QRTHLADPSYSEGMD-EYEDHMLNSSMAAILRN 378
CPSG_02828          DDFFAQGTTDLSTHRLDEAIRFAYG--QRTHMGDPRFVEGLG-EFQQDILNKSVSAQIRG 376
CIMG_05765          DDFFAQGTTDLSTHRLDEAIRFAYG--QRTHMGDPRFVEGLG-EFQQDILNKSVSAQIRG 383
CIRG_02536          DDFFAQGTTDLSTHRLDEAIRFAYG--QRTHMGDPRFVEGLG-EFQQDILNKSVSAQIRG 383
CISG_06075          DDFFAQGTTDLSTHRLDEAIRFAYG--QRTHMGDPRFVEGLG-EFQQDILNKSVSAQIRG 200
PADG_07986          DRFFSPGTVNLSTHRMTEAYRFAYG--QRTNLGDPSFVDNVT-EYEEDMLRVSTAAQIRA 400
PABG_06527          DRFFSPGTVNLSTHRMTEAYRFAYG--QRTNLGDPSFVDNVT-EYEEDMLRVSTAAQIRA 400
CIHG_09375          DDFFAQGTTDLSTHRLDEAIRFAYG--QRTHMGDPRFVEGLG-EFQQDILNKSVSAQIRG 383
CPAG_03141          DDFFAQGTTDLSTHRLDEAIRFAYG--QRTHMGDPRFVEGLG-EFQQDILNKSVSAQIRG 383
                                 :   *: :
```

```
Afu7g04760          KISDYRTQNVSAYNPQGI--ESLNEC---------EYRLAISLVTTINTLFGSQLMVPET 334
ANID_10444          KISDLRTQNVSAYDPAGI--ESLETPGTSHIATIDRSGLAVSAITTINLLFGSRIVVPET 477
UM02291             NITDDRTHPLDYYHPKFD---IIDDHGTMHLSIVDQHGMAIALTSTVNLIFGSRVMDRST 518
SPAC664.09          NISNERTFDFTHYKAEYD---FPNDHGTTHLSVIDKDNMAVGLTASINLMFGSQLLEPET 473
CNAG_01026          KITDNETHSADYYGLQYD---TPIDHGTTHLSTVDKWGGAASVTSTINLIWGSHVMDPKT 545
SPAC56E4.06c        NISLSKTYPWEHYNPSYD---LPISHGTTHVSTVDSNNLAVSITSTVNLLFGSQLMDPVT 452
Ca_orf19.6053       SDE----KTFH-WKHYDPKYDIAEPQGTSHFSVVDENDNSVAMTTTVNLLFGSMIYDSKT 473
ScYLR299W           NSQDGNFKTLENWTLYDPAYDINNPHGTAHFSIVDSHGNAVSLTTTINLLFGSLVHDPKT 502
BDCG_02331          KISDHHTQDISAYDPEGL--ESLDTPGTSHLVSTDRSGLAVSLTTTINLLFGSKLIVPET 433
BDFG_03037          KISDHHTQDISAYDPEGL--ESLDTPGTSHLVSTDRSGLAVSLTTTINLLFGSKLIVPET 433
PAAG_06130          KIFDENTQNVSAYDPAGH--QILETPGTSHLVSTDRTGLAVSLTTTINLIFGSKLMVPET 433
HCDG_09012          PTPPLKSLKLR--------------PGTSHIVAADSRGLTISLTTTINLLFGSKVMVPET 314
UM2120              QISDTRTFSASHYIPDHKNAEVKSDHGTSAITVVDADGMAISLTTTINTFWGSQLMT-EH 572
HCBG_05688          KISDSHTLNVSDYNPNGF--ESLETPGTSHIVAADSRGLTISLTTTINLLFGSKVMVPET 470
CNAG_02888          MIDENETKAPDYYKPPKV--ALEFDNGTSNITATDSSGLTISITTTVGLGWGSRIMVPGY 383
BDDG_07075          KISDHHTQDISAYDPEGL--ESLDTPGTSHLVSTDRSGLAVSLTTTINLLFGSKLIVPET 433
BDBG_06877          KISDHHTQDISAYDPEGL--ESLDTPGTSHLVSTDRSGLAVSLTTTINLLFGSKLIVPET 433
HCAG_03238          KISDSHTLNVSDYNPNGF--ESLETPGTSHVVAADSRGLTISLTTTINLLFGSKVMVPET 436
HCEG_08336          KISDSHTLNVSDYNPNGF--ESLETPGTSHIVAADSRGLTISLTTTINLLFGSKVMVPET 436
CPSG_02828          KILDERTQNISAYEPSGF--EILETPGTSHISSADRNGLAISLTTTVNLYFGSKVMVPET 434
CIMG_05765          KILDERTQNISAYEPSGF--EILETPGTSHISSADRNGLAISLTTTVNLYFGSKVMVPET 441
CIRG_02536          KILDERTQNISAYEPSGF--EILETPGTSHISSADRNGLAISLTTTVNLYFGSKVMVPET 441
CISG_06075          KILDERTQNISAYEPSGF--EILETPGTSHISSADRNGLAISLTTTVNLYFGSKVMVPET 258
PADG_07986          KIFDESTQNVSVYDPAGH--QILETPGTSHLVSTDRTGLAVSLTTTINLIFGSKLMVPET 458
PABG_06527          KIFDESTQNVSAYDPAGH--QILETPGTSHLVSTDRTGLAVSLTTTINLIFGSKLMVPET 458
CIHG_09375          KILDERTQNISAYEPSGF--EILETPGTSHISSADRNGLAISLTTTVNLYFGSKVMVPET 441
CPAG_03141          KILDERTQNISAYEPSGF--EILETPGTSHISSADRNGLAISLTTTVNLYFGSKVMVPET 441
                                                           : .  :::.  :** :
```

```
Afu7g04760          GIIMNNEMDDFSVPGKSNSFGYVPSKANYIRPGKRPLSSITPAIVTRP------DGKLFF 388
ANID_10444          GVIMNNEMDDFSVPGSSNSFGYIPSEANYIRPGKRPLSSCTPAIVTHP------NGTTFF 531
UM02291             GVILNDEMDDTSTPGVPNAFGLAPSPYNYPEAHKRPLSSTCPTIIES------ASGQVEL 572
SPAC664.09          GIILNDHMDDFASPGIVNAFGLSPSPYNFIAPGKRPQSSAVPTILV-------YNGEVEM 526
CNAG_01026          GIIFNDEQDDFAVPGAPDAFGLWPSPWNYPAPGKKPLSSTSASIILNPTTSSSPSPSLYA 605
SPAC56E4.06c        GVVFNDQMDDFSIPGASNAFNLSPSPWNFIEPFKRPQSSSAPTILTD------INGDFEM 506
Ca_orf19.6053       GIILNDEMDDFALPNVSNAFNLTPSIFNFIHPGKRPLSSTAPTIIINDATN-----STDF 528
ScYLR299W           GVIFNNEMDDFAQFNKSNSFELAPSIYNFPEPGKRPLSSTAPTIVLSELG------IPDL 556
BDCG_02331          GIIMNDEMNDFSIPNADNAFGFIPSPANFIRPGKRMLSSMCPTIVTHP------NGTLYF 487
BDFG_03037          GIIMNDEMNDFSIPNADNAFGFIPSPANFIRPGKRMLSSMCPTIVTHP------NGTLYF 487
PAAG_06130          GIVMNNEMNDFSIPDTSNKFGYIPTPANFIRPGKRMLSSMCPIIVTHP------DGSLFF 487
HCDG_09012          GIIMNNDMDDFSIPGTNNSFGYIPSPANFIEPGKRMLSSICPVIVTHP------NGTVYF 368
UM2120              GFPLNNELDDASSPGQTNFFGYLATPANYIEPGKRPLSSISAVIAEEVR-----TGELKL 627
HCBG_05688          GIIMNNDMDDFSIPGTNNSFGYIPSPANFIEPGKRMLSSICPVIVTHP------NGTVYF 524
CNAG_02888          GFVLNGSMDDFSVEGRPNGFGYEPQVTNYVAGGKRPLSSSCPYIITNS------TGKVVA 437
BDDG_07075          GIIMNDEMNDFSIPNADNAFGFIPSPANFIRPGKRMLSSMCPTIVTHP------NGTLYF 487
BDBG_06877          GIIMNDEMNDFSIPNADNAFGFIPSPANFIRPGKRMLSSMCPTIVTHP------NGTLYF 487
HCAG_03238          GIIMNNDMDDFSIPNTNNSFGYIPSPANFIEPGKRMLSSICPVIVTHP------NGTVYF 490
HCEG_08336          GIIMNNDMDDFSIPGTNNSFGYIPSPANFIEPGKRMLSSICPVIVTHP------NGTVYF 490
CPSG_02828          GIIMNNEMNDFSIPGSDNAFGYRPSPANYIRPGKRPLSSICPTIVTHP------NGTMYF 488
CIMG_05765          GIIMNNEMNDFSIPGSDNAFGYRPSPANYIRPGKRPLSSICPTIVTHP------NGTMYF 495
CIRG_02536          GIIMNNEMNDFSIPGSDNAFGYRPSPANYIRPGKRPLSSICPTIVTHP------NGTMYF 495
CISG_06075          GIIMNNEMNDFSIPGSDNAFGYRPSPANYIRPGKRPLSSICPTIVTHP------NGTMYF 312
PADG_07986          GIVMNNEMNDFSIPDTSNKFGYIPTPANFIRPGKRMLSSMCPIIVTHP------DGSLFF 512
PABG_06527          GIVMNNEMNDFSIPDTSNKFGYIPTPANFIRPGKRMLSSMCPIIVTHP------DGSLFF 512
CIHG_09375          GIIMNNEMNDFSIPGSDNAFGYRPSPANYIRPGKRPLSSICPTIVTHP------NGTMYF 495
CPAG_03141          GIIMNNEMNDFSIPGSDNAFGYRPSPANYIRPGKRPLSSICPTIVTHP------NGTMYF 495
                    *. :*.  :* :  .  : *   .   *:    *:  **  . *
```

```
Afu7g04760          LAGSAGGSRIITATVQNIIRVIDQGLTAAQALAQPRLHDQLIPNQVSFEYT--------- 439
ANID_10444          IAGSAGGSRIITATVQNIIHAVDEGLSAAEALARPRLHDQLIPNQVAFEYA--------- 582
UM02291             VLGGSGGSRIFSSVLQTIFNFYLWGMDLSQSIEAPRLHHQLLPTQLSVETG--------- 623
SPAC664.09          VLGGSGGSRIVTAVLDTIIKKYKWGKSLLESVESPRFHHQLMPNIVYIDET--------- 577
CNAG_01026          VIGGSGGSRIFPSVAQVLLNLFS-GLDISESIEAYRVHNQIVPDLTTIEVGP-------E 657
SPAC56E4.06c        ALGASGGSRIVTAVLDSIIKRIDMDYDIESMVASARPHHQLLPDILILESG--------- 557
Ca_orf19.6053       VIGAAGGSRITSAILQAIVRTYYRKYDLLSTIAFPRLHHQLIPESVMSENLTVWDQ---- 584
ScYLR299W           VVGASGGSRITTSVLQTIVRTYWYNMPILETIAYPRIHHQLLPDRIELESFPMIG----- 611
BDCG_02331          VTGAAGGSRIITSVVQSIINVIDRGMSAAEALREPRLHDQLVPNVSMFEYP--------- 538
BDFG_03037          VTGAAGGSRIITSVVQSIINVIDRGMSAAEALREPRLHDQLVPNVSMFEYP--------- 538
PAAG_06130          VTGAAGGSRIITATVQSVINVIDRKLNASRALEEPRLHDQLVPNLTLFEWA--------- 538
HCDG_09012          ITGAAGGSRIITGTVQSVINLLDRNMTTYDALKEPRVHDQLSPNVSEFEYS--------- 419
UM2120              SLSSAGGSRIITAVTQVAYNVLFKGQDAQAALAEPRWHDQLSPNQTTLESAAAAI-RGFV 686
HCBG_05688          ITGAAGGSRIITGTVQSVINLLDRNMTTYDALKEPRVHDQLSPNVSEFEYS--------- 575
CNAG_02888          SGGAAGGSTIISSNAQVAFFVLVYGFSASRALAANRLHNQILPNVTKLERGSNVRGVRVE 497
BDDG_07075          VTGAAGGSRIITSVVQSIINVIDRGMSAAEALREPRLHDQLVPNVSMFEYP--------- 538
BDBG_06877          VTGAAGGSRIITSVVQSIINVIDRGMSAAEALREPRLHDQLVPNVSMFEYP--------- 538
HCAG_03238          ITGAAGGSRIITGTVQSVINLLDRNMTTYDALKEPRVHDQLSPNVSEFEYS--------- 541
HCEG_08336          ITGAAGGSRIITGTVQSVINLLDRNMTTYDALKEPRVHDQLSPNVSEFEYS--------- 541
CPSG_02828          ITGAAGGSRIITSTLQSVINVLDRHMTAPQALAEPRLHDQLVPNLTEVEWS--------- 539
CIMG_05765          VTGAAGGSRIITSTLQSVINVLDRHMTAPQALAEPRLHDQLVPNLTEVEWS--------- 546
CIRG_02536          VTGAAGGSRIITSTLQSVINVLDRHMTAPQALAEPRLHDQLVPNLTEVEWS--------- 546
CISG_06075          VTGAAGGSRIITSTLQSVINVLDRHMTAPQALAEPRLHDQLVPNLTEVEWS--------- 363
PADG_07986          VTGAAGGSRIITATVQSVINVIDRKLNASRALEEPRLHDQLVPNLTLFEWA--------- 563
PABG_06527          VTGAAGGSRIITATVQSVINVIDRKLNASRALEEPRLHDQLVPNLTLFEWA--------- 563
CIHG_09375          VTGAAGGSRIITSTLQSVINVLDRHMTAPQALAEPRLHDQLVPNLTEVEWS--------- 546
CPAG_03141          ITGAAGGSRIITSTLQSVINVLDRHMTAPQALAEPRLHDQLVPNLTEVEWS--------- 546
                      ..:*** * ..  :               :   * *.*: *     :
```

```
Afu7g04760          -YDNSTVDFMKSRGHNVTWVAP--GQSTAQAIRVLPNG----TFDAAGEPR--------- 483
ANID_10444          -YDNSTVAFMAARGHNVTWMAP--GTSTAQAIRVLPNG----TFEAAGEPR--------- 626
UM02291             -YSEKVLSGLLGRGHEVSWIDIDLGIAEVQAVAVQAEGKRWKRVWAASDSR--------- 673
SPAC664.09          -VEIEVLRALEKFGHIVD-LIPVQYPFSEIQAVFRTNG----TLYGLSDSR--------- 622
CNAG_01026          GVDEEVVEGLKERGHKIGEFDVNIGISEVQAIVVEDG-----HIFASSDSR--------- 703
SPAC56E4.06c        -FSKSVATRMKKYGHKVWRLKQHDTPLSQIQAVTRHHS----EYYGMSDPR--------- 603
Ca_orf19.6053       -EHTGIASSMKKLGHTFLETGS---LTAMNGIKRVKGG----KLHGVSDWWR-------- 628
ScYLR299W           ---KAVLSTLKEMGYTMKEVFP---KSVVNAIRNVRG-----EWHAVSDYWR-------- 652
BDCG_02331          -FDNSTVAFMAGRNHTVQHVPL--GRSTVQSIRVFGES----KFEAVGEPR--------- 582
BDFG_03037          -FDNSTVAFMAGRNHTVQHVPL--GRSTVQSIRVFGES----KFEAVGEPR--------- 582
PAAG_06130          -FDNSTVEFMAERGHNVTRVAP--GFSSVQCIQVLGNS----TFEAVGEPR--------- 582
HCDG_09012          -FDNSTVAFMISRNHSVKWVLP--GASKVQAIRLMADG----MYEAVGEPR--------- 463
UM2120              GFNNQTAAFLASVGHNVSWTPI--GSSTAQAVERFPDG----VLLAATETRSSLMLILVK 740
HCBG_05688          -FDNSTVAFMISRNHSVKWVLP--GASKVQAIRLMADG----MYEAVGEPR--------- 619
CNAG_02888          GFSEEQVKGLKQKGHVIEWVDK--SFSTP-CVMVWTDD----DWEGDGDPR--------- 541
BDDG_07075          -FDNSTVAFMAGRNHTVQHVPL--GRSTVQSIRVFGES----KFEAVGEPR--------- 582
BDBG_06877          -FDNSTVAFMAGRNHTVQHVPL--GRSTVQSIRVFGES----KFEAVGEPR--------- 582
HCAG_03238          -FDNSTVAFMISRNHSVKWVLP--GASKVQAIRLMADG----MYEAVGEPR--------- 585
HCEG_08336          -FDNSTVAFMISRNHSVKWVLP--GASKVQAIRLMADG----MYEAVGEPR--------- 585
CPSG_02828          -FDNSTVEFLIDRGHNITRVEH--ASSTVQSIRVLSNG----TFEAAGEPR--------- 583
CIMG_05765          -FDNSTVAFLIDRGHNITRVEH--ASSTVQSIRVLSNG----TFEAAGEPR--------- 590
CIRG_02536          -FDNSTVAFLIDRGHNITRVEH--ASSTVQSIRVLSNG----TFEAAGEPR--------- 590
CISG_06075          -FDNSTVAFLIDRGHNITRVEH--ASSTVQSIRVLSNG----TFEAADLVF--------- 407
PADG_07986          -FDNSTVEFMAERGHNVTPVAP--GFSSVQCIQVLGNS----TFEAVGEPR--------- 607
PABG_06527          -FDNSTVEFMAERGHNVTPVAP--GFSSVQCIQVLGNS----TFEAVGEPR--------- 607
CIHG_09375          -FDNSTVAFLIDRGHNITRVEH--ASSTVQSIRVLSNG----TFEAAGEPR--------- 590
CPAG_03141          -FDNSTVEFLIDRGHNITRVEH--ASSTVQSIRVLSNG----TFEAAGEPR--------- 590
                             :   .: .                            .
```

```
Afu7g04760          ------QLDSGGFSI--------------------------------------------- 492
ANID_10444          ------QLDAGGFAV--------------------------------------------- 635
UM02291             ---------KGGVAVAV------------------------------------------- 681
SPAC664.09          ---------KQAVAAAY------------------------------------------- 630
CNAG_01026          ---------KNGIAAGY------------------------------------------- 711
SPAC56E4.06c        ---------KYGQAAAY------------------------------------------- 611
Ca_orf19.6053       ---------KRGESDGY------------------------------------------- 636
ScYLR299W           ---------KRGISSVY------------------------------------------- 660
BDCG_02331          ------QKNSGGVVVCGGECADFC------------------------------------ 600
BDFG_03037          ------QKNSGGVVVCGGECADFC------------------------------------ 600
PAAG_06130          ------QKNSGGIVV--------------------------------------------- 591
HCDG_09012          ------QVDSGGAVA--------------------------------------------- 472
UM2120              VVFQFWQFSSKCTITSWEPTTTFTALVRGLAECRQRADLYGTLTSRPFERRAYLASDARR 800
HCBG_05688          ------QVDSGGAVA--------------------------------------------- 628
CNAG_02888          ------KHDSGGSVYHEY------------------------------------------ 553
BDDG_07075          ------QKNSGGVVVCGGECADFC------------------------------------ 600
BDBG_06877          ------QKNSGGVVVCGGECADFC------------------------------------ 600
HCAG_03238          ------QVDSGGAVA--------------------------------------------- 594
HCEG_08336          ------QVDSGGAVA--------------------------------------------- 594
CPSG_02828          ------QLNSGGLAI--------------------------------------------- 592
CIMG_05765          ------QLNSGGLAI--------------------------------------------- 599
CIRG_02536          ------QLNSGGLAI--------------------------------------------- 599
CISG_06075          ------LRLEFGRDHDRT------------------------------------------ 419
PADG_07986          ------QKNSGGIVV--------------------------------------------- 616
PABG_06527          ------QKNSGGIVV--------------------------------------------- 616
CIHG_09375          ------QLNSGGLAI--------------------------------------------- 599
CPAG_03141          ------QLNSGGLAI--------------------------------------------- 599
```

```
Afu7g04760          ------------------------------------------------------------
ANID_10444          ------------------------------------------------------------
UM02291             ------------------------------------------------------------
SPAC664.09          ------------------------------------------------------------
CNAG_01026          ------------------------------------------------------------
SPAC56E4.06c        ------------------------------------------------------------
Ca_orf19.6053       ------------------------------------------------------------
ScYLR299W           ------------------------------------------------------------
BDCG_02331          ------------------------------------------------------------
BDFG_03037          ------------------------------------------------------------
PAAG_06130          ------------------------------------------------------------
HCDG_09012          ------------------------------------------------------------
UM2120              VRVLRDARLSLRMIEWRLDNISLPMLQHSDDDDLRVQRWPIKQDKTQASNSGFFHSWLVK 860
HCBG_05688          ------------------------------------------------------------
CNAG_02888          ------------------------------------------------------------
BDDG_07075          ------------------------------------------------------------
BDBG_06877          ------------------------------------------------------------
HCAG_03238          ------------------------------------------------------------
HCEG_08336          ------------------------------------------------------------
CPSG_02828          ------------------------------------------------------------
CIMG_05765          ------------------------------------------------------------
CIRG_02536          ------------------------------------------------------------
CISG_06075          ------------------------------------------------------------
PADG_07986          ------------------------------------------------------------
PABG_06527          ------------------------------------------------------------
CIHG_09375          ------------------------------------------------------------
CPAG_03141          ------------------------------------------------------------
```

```
Afu7g04760          --
ANID_10444          --
UM02291             --
SPAC664.09          --
CNAG_01026          --
SPAC56E4.06c        --
Ca_orf19.6053       --
ScYLR299W           --
BDCG_02331          --
BDFG_03037          --
PAAG_06130          --
HCDG_09012          --
UM2120              LS 862
HCBG_05688          --
CNAG_02888          --
BDDG_07075          --
BDBG_06877          --
HCAG_03238          --
HCEG_08336          --
CPSG_02828          --
CIMG_05765          --
CIRG_02536          --
CISG_06075          --
PADG_07986          --
PABG_06527          --
CIHG_09375          --
CPAG_03141          --
```

Supp. Fig. 2. Domains of secreted glutathione-dependent ferric reductases from different fungi. First, amino acid sequences from different fungi were obtained in the respective genome database and aligned using ClustalX. In sequence, protein domains were identified in Ggt1 homologs using SMART online tool (http://smart.emblheidelberg.de/): gamma-glutamyltranspeptidase domain (in bold), transmembrane domain (in italic) and signal peptide (grey box). *Paracoccidioides* spp. proteins are highlighted in bold red. Asterisks indicate amino acid identity and dots represent conserved substitutions. Afu: *A. fumigatus*; ANID: *A. nidulans*; Ca: *C. albicans*; CNAG: *C. neoformans*; Sc: *S. cerevisiae*; SPAC: *S. pombe*; UM: *U. maydis*; CI: *C. immitis*; CP: *C. posadasii*; PAAG: *Pb*01; PADG: *Pb*18; PABG: *Pb*03; BD: *B. dermatitidis*; HC: *H. capsulatum*.
