# Supplementary material for: Paracoccidioides spp. ferrous and ferric iron assimilation pathways
Source: Front Microbiol. 2015 Aug 12;6:821. doi: 10.3389/fmicb.2015.00821 (PMC4585334; doi:10.3389/fmicb.2015.00821)
Supplement: Supplementary file 5 [file Image3.PDF]

```
PAAG_03681              ---------------------------------------------MLPSFSFFLTWFIFL 15
PADG_03184              ---------------------------------------------MLLSFSFFLTWFIFL 15
PAAG_00163              ------------------------------------------------------------
PADG_07092              --------------------------------------------------MIPSRRKARL 10
PAAG_06004              MERPSSRRRIRGPRNPQECRRESPGSREGTPGHGTVHWSKRHLAVVVISVISLAALFRVL 60
PADG_05994              MERPSSRRRIRGARNSQECRRVSPGSGEGKPSQGTVHWSKRHLAVVVISVISLVALFRVL 60
ScYMR058W_Fet3          -------------------------------------------------MTNALLSIAVL 11

PAAG_03681              PQVALAAQCK-----------------------PHSPGVRRFELELTWVTAS----PDGV 48
PADG_03184              PQAALAGQCK-----------------------PYSPGVRRFELELTWITAS----PDGV 48
PAAG_00163              --------------------------------------------------MA----PDGF 6
PADG_07092              DGSEIKIANT-----------------------AHQSDPAGYHLEISNTTMA----PDGF 43
PAAG_06004              EGSNIPGYLPGLALKDHHVLDVSLHPATEELHPEDHIYRRAKTIHLDWTVTAGDRHPDGV 120
PADG_05994              EGSNIPGYLPGLALKEH-VLDVSLHPATEELHPEDHIYRRATTIHLNWTVTAGDRHPDGV 119
ScYMR058W_Fet3          LFSMLS-------------------------------LAQAETHTFNWTTGWDYRNVDGL 40
                                                                                 **.

PAAG_03681              E-RQMIFTNNQFPGPQLDVIEGDKVEVVVKNSLP-FSTAIHFHGISQKGTPWSDGVPDVT 106
PADG_03184              E-RQMIFTNNQFPGPQLDIIEGDKVEVVVKNSLP-FSTAIHFHGISQKGTPWSDGVPDVT 106
PAAG_00163              E-RLVLAVNGQFPGPIIRA----------KQAYIGTEFGRSIQVIKMAQTGSQN-ANSRC 54
PADG_07092              E-RLVLAVNGQFPGPIIRANWGDTLRIHVKNSLQNNGTSIHWHGIRQKYTSHQDGTNGVT 102
PAAG_06004              R-KSVYLINGLFPGPTIEARSGDRLIINVKNGLADEGVSVHWHGLHMKDGNRMDGTTGVT 179
PADG_05994              R-KSVYLINGRFPGPTIEARSGDRLIINVKNGLADEGVSVHWHGLHMKDGNRMDGTTGVT 178
ScYMR058W_Fet3          KSRPVITCNGQFPWPDITVNKGDRVQIYLTNGMNNTNTSMHFHGLFQNGTASMDGVPFLT 100
                        . : :   *. ** * :            .:.      .   : :        : .

PAAG_03681              QRAIQPGKSFIYRWTAV--EYGTYWHHGHVHGQVSDGLFGAIVIHPAKGRLAPFGKISSS 164
PADG_03184              QRAIQPGKSFIYRWTAV--EYGTYWYHGHEHGQVSDGLFGAIVIRPAKDRLAPFGKISSS 164
PAAG_00163              IGPIAPGATKTYEFKCT--QYGTSWYHSHHTVQYGDGVLGPIIIDGP------------- 99
PADG_07092              ECPIAPGATKTYEFKCT--QYGTSWYHSHHTVQYGDGVLGPIIIDGP------------- 147
PAAG_06004              QCAIAPEESFPYDFTISDSQSGTYWYHAHSGLQRADGLYGGLVIHRPAPRG--VRGIQLR 237
PADG_05994              QCAIAPQESFLYDFTISDSQSGTYWYHAHSGLQRADGLYGGLVIHRPAPRG--VRGVQLR 236
ScYMR058W_Fet3          QCPIAPGSTMLYNFTVD-YNVGTYWYHSHTDGQYEDGMKGLFIIKDD------------- 146
                          .* *  :  * :.    : **  *:*.*   *  **: * ::*

PAAG_03681              AKDLQSIKKAELKPIPIFLSDWHHLTAAEYFNVELESG-IDN--FCSDSILINGKGSVIC 221
PADG_03184              AKDLQSIKKAELKPIPIFLSDWHHLTAAEYFNVELESG-IDN--FCSDSILINGKGSVIC 221
PAAG_00163              -----ATANYDIDLGTMPIQDWYYRTAWQNALIGIPPI-AEN--GLLNGTMVNAKG---- 147
PADG_07092              -----ATANYDIDLGTMPIQDWYYRTAWQNALIGIPPI-AEN--GLINGTMVNAKG---- 195
PAAG_06004              HTESDILRYNYQKEILLLVGDWYHRRAHDVLEWYMRAG-SYGNEPVPDSLVINGAGHFNC 296
PADG_05994              HPESDILRYNYQKEILLLVGDWYHRRAHDVLEWYMRAG-SYGNEPVPDSLVINGAGHFNC 295
ScYMR058W_Fet3          -----SFPYDYDEELSLSLSEWYHDLVTDLTKSFMSVYNPTGAEPIPQNLIVNNTMN--- 198
                                    .   : : :*::  . :     :      .     :. ::*

PAAG_03681              KTQDEVNRLARRDQSMLLVNETLTDKGCLPYYLPSVVGDFPIVPEKVPKDLFYNCHPSHG 281
PADG_03184              KTQDEVNRLTRQDQSVLLVNETLTDKGCLPYYLPSVVGDFPIVPEKVPKDLFYNCHPSHG 281
PAAG_00163              ----------------------------------------------------------GG 149
PADG_07092              ----------------------------------------------------------GG 197
PAAG_06004              AQAVPARP------------------------------------------VDCLGDGH 312
PADG_05994              AQAVPARP------------------------------------------VDCLEDGH 311
ScYMR058W_Fet3          ------------------------------------------------------------

PAAG_03681              EHEVITVNPADGWASLNFINSATIATFMLSIDEHKMWVYAVDG--------HYIDPVLVD 333
PADG_03184              EHEVITVDPADGWASLNFINSATIATFMLSIDEHKMWVYAVDG--------HYIDPVLVD 333
PAAG_00163              SYHQNTIKKGKSYR-LRLINTSVDNYFKVHLDNHTFTVISSDF--------VPIVPYQAD 200
PADG_07092              RYHKNTIKKGKSYR-LRLINTSVDNYFKVHLDNHTFTVISSDF--------VPIVPYQAD 248
PAAG_06004              PVPYLIIDPTQSYR-VRLVNTGSLAGISLGVAQGYLDVLHLDGGLGVQQLRQSKTSNPKS 371
PADG_05994              PVPYLIIDPTQSYR-VRLVNTGSLAGISLGVAQGYLDVLHLDGGLDVQELRRPKTSNPQS 370
ScYMR058W_Fet3          --LTWEVQPDTTYL-LRIVNVGGFVSQYFWIEDHEMTVVEIDG--------ITTEKNVTD 247
                              :.    :  :.::* .      . : :  : *   *                 .
```

```
PAAG_03681          GIPIANGNRFSALVRLDK---PRRDYTIRAANVASSQILSGFGTLRYKGQDKNIPRKPSK 390
PADG_03184          GIPIANGNRFSALVRLDK---PRGDYTIRAANVASSQIVSGFGTLRYKGQDTNNPRKPSK 390
PAAG_00163          WLFIGIGQRYDVIIHADQ---DIGNYWFRAEVQQGCGLNAMNGRIRSIFRYEGADEKALP 257
PADG_07092          WLFIGIGQRYDVIIHADQ---DIGNYWFRAEVQQGCGINAMNGRIRSIFRYEGADEQALP 305
PAAG_06004          IGILYPGQRVDFILRHHRNAPAESSLTVELDPECFKYSNPALSSLQSFPIYSTISNSIKQ 431
PADG_05994          IGILYPGQRVDFILRHHRNAPAKSSLTVELDPECFKYSNPALSSLQSFPIYSTISKSIKQ 430
ScYMR058W_Fet3      MLYITVAQRYTVLVHTKN-DTDKNFAIMQKFDDTMLDVIPSDLQLNATSYMVYNKTAALP 306
                       :  .:*   ::: ..        ..          .    :.

PAAG_03681          PSIDYNGLNLTADFVALEENKIVPFPPS---KPAAKADTTYKFDFGFFSSAFRWTVSGEA 447
PADG_03184          PSIDYNGLNLTADFVALEENKIVPFPPS---KPAAKADTTYKFNFGFFDSAFRWTVSGEA 447
PAAG_00163          TSIASNYTQSCTDEQGLVPFVPINVPSAHFIQHVKDLNVTFESEYSSPGNNFVHWRINGI 317
PADG_07092          TSIASNYTQSCTDERGLVPFVPISVPSAHFTQHVKDLNVTFESEYSSPGNNFVHWRINGI 365
PAAG_06004          TSLPQPLTLPAKNHIDLTQVSSTPALLSGLPPEADQTYVVYTKISKMSKNQNVPLGYFNQ 491
PADG_05994          TSLPQPLTLPAKNHIDLTQVSSTPALLSGLPPEADQTYVVYTKISKMSKNHNVPLGYFNH 490
ScYMR058W_Fet3      TQNYVDSIDNFLDDFYLQPYEKEAIYGE--PDHVITVDVVMDNLKNGVNYAFFNNITYTA 364
                    ..          :   *                * .    ..

PAAG_03681          SLNISQGVKPVLFDING--PLGSNRNLTFPTKNGTWVDLILITDG--IFNPPHPIHKHSN 503
PADG_03184          SLNISQGIKPVLFDING--PLGSHRNLTFPTKNDTWVDLILITGG--TFNPPHPIHKHSN 503
PAAG_00163          PIETDWEYPTLQYVLDGNTSYPRKLNLIELPEHNVWTYWIIQAAFGSIVNVPHPIHLHGH 377
PADG_07092          PIETDWEYPTLQYVLDGNTSYPRKLNLIELPEHNVWTYWIIQAAFGSIVNVPHPIHLHGH 425
PAAG_06004          TSWRPQSTLRYPLIALDPHDWDKNQFAISTGSKPVWVDLVINNLD----EGAHPFHLHGH 547
PADG_05994          TSWRPQSTPRYPLIALDPHDWDKNQLAISTGSKPVWVDLVINNLD----EGAHPFHLHGH 546
ScYMR058W_Fet3      PKVPTLMTVLSSGDQANNSEIYGSNTHTFILEKDEIVEIVLNNQD----TGTHPFHLHGH 420
                    .               .              .:   .  ::         .**:* *.:

PAAG_03681          KVYLIGKGTGPFPWDTV------EDAIKEMPQFFNLETPRFVDGFTSPPGTKQNQGWVAV 557
PADG_03184          KVYVIGKGTGPFPWDTV------EDAIKEMPQFFNLETPRFVDGFTSPPGTKLNPGWVAV 557
PAAG_00163          DFSILGTGVGDFPG----------------PNVLNFTNPPRRDVAMLP-----DLGWLAI 416
PADG_07092          DFSILGTGVGDFPG----------------PNSLNFTNPPRRDVAMLP-----DLGWLAI 464
PAAG_06004          NFFVLTLHAATQGWGSYNPFDPHPRHQQFRPSSKDLAKAVLRDTVQIP-----QRGHAVL 602
PADG_05994          DFFVLTLHAATQGWGSYNPFDPHPRHQQFRPSSKDLEKAVLRDTVQIP-----QRGHAVL 601
ScYMR058W_Fet3      AFQTIQRDRTYDDALGEVP-----HSFDPDNHPAFPEYPMRRDTLYVR-----PQSNFVI 470
                     .  :                                 .   *            .  .:

PAAG_03681          RYHVVNPGPFLLHCHIQTHFTGGMGIVLLDG---------------------VDKLPEVP 596
PADG_03184          RYHVVNPGPFLLHCHIQTHFAGGMGIVLLDG---------------------VDKLPKVP 596
PAAG_00163          AFPTDNPGAWLAHCHIAWHAHEGLAVQFLER---------------------GSNILSAL 455
PADG_07092          AFPTDNPGAWLAHCHIAWHAHEGLAVQFLEQ---------------------GSNILSAL 503
PAAG_06004          RFRADNPGIWLLHCHILWHLAAGMAMVIDVMN--------------------GFNVGEKP 642
PADG_05994          RFRADNPGVWLLHCHILWHLAAGMAMVIDVMN--------------------GFNVGEKP 641
ScYMR058W_Fet3      RFKADNPGVWFFHCHIEWHLLQGLGLVLVEDPFGIQDAHSQQLSENHLEVCQSCSVATEG 530
                     : . *** :: ****  *   *:.: :                          .:

PAAG_03681          -------------LEYLKAEF--------------------------------------- 604
PADG_03184          -------------LEYLKAEF--------------------------------------- 604
PAAG_00163          NIRPEWETTCRQWRDYISSRALFQQDRSGV------------------------------ 485
PADG_07092          NIRPEWETTCRQWRDYISNSALFQQDRSGV------------------------------ 533
PAAG_06004          EIAG---------GENIAMCQYF------------------------------------- 656
PADG_05994          GIAG---------GENIAMCQYF------------------------------------- 655
ScYMR058W_Fet3      NAAANTLDLTDLTGENVQHAFIPTGFTKKGIIAMTFSCFAGILGIITIAIYGMMDMEDAT 590
                                  : :

PAAG_03681          ----------------------------------------------
PADG_03184          ----------------------------------------------
PAAG_00163          ----------------------------------------------
PADG_07092          ----------------------------------------------
PAAG_06004          ----------------------------------------------
PADG_05994          ----------------------------------------------
ScYMR058W_Fet3      EKVIRDLHVDPEVLLNEVDENEERQVNEDRHSTEKHQFLTKAKRFF 636
```

Supp. Fig. 3. Alignment of amino acid sequences of *Sc*Fet3 and *Paracoccidioides* spp. MCOs. First, amino acid sequences were obtained in the respective genome database and

aligned using ClustalX. Ferroxidase motifs (in bold), including the Cu-ligands (in bold red), and residues involved in Fe^{2+} binding (in bold yellow) (Kosman, 2010) were highlihted. Moreover, protein domains were identified using SMART online tool (http://smart.emblheidelberg.de/): signal peptide (in italic) and transmembrane region (underlined). Asterisks indicate amino acid identity and dots represent conserved substitutions. Sc: *S. cerevisiae*; PAAG: *Pb*01; PADG: *Pb*18.
